# Association between coffee consumption and life expectancy: a prospective cohort study from NHANES 2001–2018

**DOI:** 10.1017/S1368980025100888

**Published:** 2025-08-22

**Authors:** Guangcan Yan, Xiaoqi Dai, Yun Yan, Jie Yan, Wei Tian, Rui Jiang

**Affiliations:** 1 School of Public Health, Harbin Medical University, Harbin, People’s Republic of China; 2 School of Public Health, Zunyi Medical University, Zunyi, People’s Republic of China; 3 Department of Pain Management, Second Affiliated Hospital of Harbin Medical University, Harbin, People’s Republic of China; 4 Department of Forensic Medicine, Guizhou Medical University, Guiyang, People’s Republic of China; 5 Department of Ultrasound, The Second Affiliated Hospital of Zunyi Medical University, Zunyi, People’s Republic of China; 6 Department of Cell Biology, Harbin Medical University, Harbin, People’s Republic of China; 7 Department of General Practice, The Second Affiliated Hospital of Harbin Medical University, Harbin, People’s Republic of China

**Keywords:** Coffee consumption, Life expectancy, Life gain, Mortality

## Abstract

**Objective:**

To assess the association between coffee consumption and life expectancy among the US adults.

**Design:**

Prospective cohort.

**Setting:**

National representative survey in the United States, 2001–2018.

**Participants:**

A total of 43 114 participants aged 20 years or older with complete coffee consumption data were included from National Health and Nutrition Examination Survey 2001–2018.

**Results:**

Over a median follow-up of 8·7 years, 6234 total deaths occurred, encompassing 1929 deaths from CVD and 1411 deaths from cancer. Based on the nationally representative survey, we found that coffee consumption is associated with longer life expectancy. The estimated life expectancy at age 50 was 30·06 years (95 % CI, 29·68, 30·44), 30·82 years (30·12, 31·57), 32·08 years (31·52, 32·70), 31·24 years (30·29, 32·19), and 31·45 years (30·39, 32·60) in participants consuming 0, ≤ 1, 1 to ≤ 2, 2 to ≤ 3, and > 3 cups of coffee per day, respectively. Consequently, compared with non-coffee drinkers, participants who consumed 1 to ≤ 2 cups/day had a gain of 2·02 years (1·17, 2·85) in life expectancy on average, attributable to a 0·61-year (29·72 %) reduction in CVD deaths. Similar benefits were found in both males and females.

**Conclusion:**

Our findings suggest that moderate coffee consumption (approximately 2 cups per day) could be recommended as a valuable component of a healthy diet and may be an adjustable effective intervention measure to increase life expectancy.

The United States (US) leads the total healthcare spending among countries, ranking first both in absolute terms (with a per capita expenditure of $11 582) and as a percentage of Gross Domestic Product (17·7 %) in 2019^(1)^. However, this substantial expenditure does not consistently contribute to better health outcomes, with shorter life expectancy (LE) lagging behind other developed nations, ranked 40th in the world for LE at birth^(2)^. Chronic diseases stand as the primary cause of premature mortality, contributing significantly to tremendous healthcare expenditures in the US^(3,4)^. Fortunately, most chronic diseases can be prevented by living a healthy lifestyle. Coffee consumption, as an adjustable healthy lifestyle, has been widely researched to be beneficial to a spectrum of health outcomes, including mortality and various chronic diseases, such as CVD, cancer, and diabetes^(5–11)^. Of course, coffee consumption is not suitable for everyone and may pose potential health risks for individuals with caffeine metabolism disorders or heightened nervous system sensitivity.

Although numerous studies have indicated that coffee consumption is linked to a decreased risk of mortality, its effect on LE remains unclear. Compared with mortality risk, LE has emerged as a more accessible metric for both policymakers and the public, providing straightforward insights into the absolute quantitative assessment of lifespan. To narrow the gap in LE between the US and other developed countries, some studies explored the effects of different risk factors on LE^(12–16)^. However, exploring multiple combined risk factors in these studies complicates the implementation of specific intervention and prevention measures given the challenge and complexity of concurrently altering multiple risk factors. Coffee consumption, an easily modifiable lifestyle factor, is considered a practical and effective intervention tool. Being the most commonly consumed beverage in the US, even small individual health effects may have profound implications on a population scale^(5)^. However, to our knowledge, research on the effect of coffee consumption on LE in the US population is lacking.

Utilizing data from the National Health and Nutrition Examination Survey (NHANES), our objective was to assess the effect of coffee consumption on mortality risk, LE, and the years of life gain attributed to coffee consumption in US adults.

## Methods

### Study design and population

The NHANES is a nationally representative survey conducted by the National Center for Health Statistics to assess the health and nutritional status of US population. Study participants were enrolled using a complex, stratified, multistage probability survey design with data release in 2-year cycles. They were invited to complete personal interviews at home and conduct physical examinations and laboratory tests at a mobile examination center. All data were collected using standard questionnaires and protocols^(17)^. To ensure nationally representative estimates, all analyses incorporated dietary sampling weights appropriately. The NHANES protocols received approval from the Ethics Review Board at the National Center for Health Statistics. All participants provided written informed consent.

We used data from nine cycles of continuous NHANES from 2001–2002 to 2017–2018, which provided detailed information on coffee consumption. The present study included 44 501 participants aged 20 years or older, all of whom had complete data on coffee consumption at baseline. After excluding pregnant women (*n* 1146) and those with incomplete data of mortality (*n* 79), smoking (*n* 29), education status (*n* 40), marital status (*n* 15), and physical activity (*n* 78), 43 114 participants were eligible for this study (see online supplementary material, Supplemental eFigure 1). Pregnant women were excluded from the study because their health status and dietary habits may differ significantly from those of the general population, and coffee consumption may have distinct health effects on both the mother and the developing fetus.

### Assessment of coffee consumption

Coffee consumption was assessed by trained interviewers using 24-hour dietary recalls with automated multiple-pass methods^(17,18)^. A standardized collection of measuring tools, such as cups, spoons, and bowls, was employed to assist the participants in reporting the size and measurements of consumed food items. The consumption of all coffee varieties within the preceding 24 h (from midnight to midnight) was documented. Before 2002, only one 24-hour dietary recall interview was conducted in-person at a mobile examination center. From 2003 onward, a second interview was conducted via telephone approximately 3–10 d after the first. Therefore, data from single-day participants were used as documented, whereas data from the 2-day participants were averaged. In a subset of 28 866 participants who completed two 24-hour recall interviews, the weighted correlation coefficient (*r*) between first and second 24-hour recall coffee consumption was 0·74 (95 % CI, 0·72, 0·75).

Based on the food codes provided by United States Department of Agriculture, which include detailed information on coffee consumption, we excluded the consumption of dry coffee powder because it was difficult to determine the equivalent number of cups in coffee beverages (eTable 1). For every participant, we merged consumption of all kinds of coffee beverage (all food codes) into a continuous value and then grouped it based on a standard cup size (8 oz or 236·56 mL) into five categories: 0, ≤ 1 cup (low), 1 to ≤ 2 cups (moderate), 2 to ≤ 3 cups (high), and > 3 cups (very high) per day. We further categorized coffee consumption into groups based on the caffeine status (caffeinated, decaffeinated), preparation method (instant coffee and non-instant coffee), and whether sugar was added into coffee (with or without sugar). Groups in caffeine status and preparation method were directly categorized by description of detailed food codes. The ‘With sugar’ group was defined as having added sugar in coffee higher than zero grams according to the Food Patterns Equivalents Database, which converts foods and beverages into 37 Food Patterns components, including added sugar.

### Ascertainment of mortality

Data from the NHANES Linked Mortality, updated on December 31, 2019, were utilized to identify the mortality status of participants. The follow-up period was determined from the survey interview date until the occurrence of death or the end of follow-up, whichever came first. The Tenth Revision of the International Classification of Diseases was used to identify causes of death. Then, we identified the primary causes of death, including fatalities attributed to CVD (I00–I09, I11, I13, and I20–I51), malignant neoplasms (C00–C97), and other causes.

The all-cause mortality rate of the population in 2019 by single-year age was obtained from the National Center for Health Statistics and further delineated based on sex, race, and ethnicity, as applicable. For CVD mortality rates, as the data only extended up to 84 years, we employed Poisson regression models with linear and squared terms of age to project mortality rates for ages beyond 84 (see online supplementary material, Supplemental eFigure 2)^(13)^.

### Covariates

To mitigate potential confounding effects, we adjusted for the following covariates in our regression models. Sociodemographic details encompassing age, sex, race and ethnicity (White, Black, Hispanic (including Mexican American, other Hispanic), and others (including Asians or multiracial)), family income, marital status, educational attainment and behavioral factors, including smoking and physical activity, were obtained using standardized questionnaires^(17)^. Sufficient physical activity was defined as ≥ 150 min of light to moderate intensity activity each week, or ≥ 75 min of vigorous-intensity activity, or an equivalent combination. Family income was evaluated by the value of family income divided by official poverty threshold and categorized as low (≤ 1·30), moderate (1·31–3·50), and high (> 3·50). For covariates with fewer missing values (including smoking, education status, marital status, and physical activity), samples with missing values were directly excluded. However, since more than 7 % of participants had missing values for family income, we treat missing values as a separate category for analysis. At one sensitivity analysis, we repeated the analyses after imputing the missing values. Dietary intake variables, including the consumption of alcohol, tea, fruits, grains, vegetables, red meat, and dairy, were collected using 24-hour dietary recalls^(17,18)^. Considering the disparities in LE between males and females, we provided detailed baseline characteristics by sex.

### Statistical analysis

We constructed Cox proportional hazards models to assess the effect of coffee consumption on the risk of all-cause and cause-specific mortality, adjusting all aforementioned covariates, which were potentially affecting the association. Linear trends of hazard ratio were examined by treating each level of coffee consumption as a continuous value in the regression models. Nonlinear trends were tested by adding a quadratic term to the models. Both linear and nonlinear trends were tested and if the *P*-value for the nonlinear trend was less than 0·05, it suggested the presence of a nonlinear trend, regardless of whether the linear trend is statistically significant or not. The Kaplan-Meier and Schoenfeld residual methods were employed to test the proportional hazards assumption, and no violation was observed. Restricted cubic spline was used to explore the nonlinear association between coffee consumption and all-cause mortality. Considering the potentially different effects of sex, race and ethnicity, and coffee categories, we conducted stratified analyses according to sex (female and male), ethnicity (White, Black, and Hispanic), caffeine status (caffeinated, decaffeinated), preparation method (instant and non-instant), and sugar addition (with sugar and without sugar). In subgroup analyses by race and ethnicity, due to limited sample size and the absence of mortality rate data for ‘others’ group of ethnicity, the ‘others’ group was directly excluded from subgroup analysis. To assess potential interactions between coffee consumption and sex or race and ethnicity, we evaluated the *P*-values for interaction by incorporating interaction terms into Cox models.

We used the life table method to estimate the LE of participants consuming different levels of coffee. The life table was built from the ages of 50 to 100 years with three estimates: (1) mortality rates of all-cause, CVD, and cancer in 2019 (mortality rates by sex and race were used as appropriate) by age; (2) overall and sex- and race-specific adjusted hazard ratios (HRs) of all-cause mortality in different levels of coffee consumption *v*. no consumption; and (3) population prevalence in age groups with intervals of 5 years, starting from 20 years old. Detailed information on the estimation of the expected survival time has been provided in previous studies^(12,13)^. Life gain or loss attributed to coffee consumption was calculated as the difference in life expectancies between the reference and exposure groups. Confidence intervals (CIs) for LE were estimated using Monte Carlo simulation with 1000 runs. To identify the leading drivers for the life gain from coffee consumption, Arriaga decomposition method was used^(19,20)^. The decomposition process involves two dimensions: first, by age group with 1-year interval and subsequently by cause of death in each age group.

We conducted an array of sensitivity analyses to evaluate the reliability of our findings. First, the participants with missing covariate values were excluded from the analysis. Second, we specifically focused on individuals who consumed coffee, using those who drank ≤ 1 cup as the reference group. Third, we repeated the analyses after imputing missing covariate data with multiple imputation by with chained equations^(21)^. Fourth, to alleviate the influence of reverse causality, participants who experienced mortality within the 2-year follow-up period were excluded. Fifth, we included the presence of at least one of the following chronic conditions–diabetes, hypertension, or dyslipidemia (definition provided in the online supplementary material, Supplemental File)–as a covariate to assess the potential impact of these common chronic diseases on the association between coffee consumption and all-cause and cause-specific mortality^(22,23)^. Last, in addition to including the presence of the three aforementioned diseases, we also replaced all diet-related variables with the HDPI (Healthy Plant-Based Diet Index; definition provided in the online supplementary material, Supplemental File) to evaluate the influence of dietary factors on the association between coffee consumption and all-cause and cause-specific mortality. All statistical analyses were conducted in R (version 4.2.2). Two-sided statistical tests were employed for all analyses, and statistical significance was defined as *P* < 0·05.

## Results

### Basic characteristics of participants

A total of 43 114 participants, representing more than 215 million adults in the US, were included in our analysis, and more than half of them reported drinking coffee (Table 1). At baseline, those who did not drink coffee were more likely to be younger, Black, non-smokers, and unmarried. Participants with higher coffee consumption were more likely to be male, White, smokers, and with higher levels of educational attainment and income, than those with lower coffee consumption. The detailed basic characteristics by sex were presented in eTable 2 and eTable 3. Similar characteristics were found in both males and females, while males were more likely to drink alcohol, consume grains and red meat, smoke, have sufficient physical activity, and have a higher level of family income.

**Table 1.** Characteristics of study participants in NHANES by different levels of coffee consumption^*^

Variable	Total		0 cup		≤ 1 cup		1 to ≤ 2 cups		2 to ≤ 3 cups		> 3 cups	
No. of participants	43 114		19 287		5867		9679		4668		3613	
	*n*	%	*n*	%	*n*	%	*n*	%	*n*	%	*n*	%
Weighted prevalence, % (weighted *n*, in millions)	100	215·4	43·7	94·2	11·0	23·7	22·0	47·4	12·5	27·0	10·8	23·2
	Mean	se	Mean	se	Mean	se	Mean	se	Mean	se	Mean	se
Age, years (se)	47·4	0·2	43·0	0·3	51·8	0·4	50·9	0·3	51·0	0·3	49·6	0·3
Tea, servings/d (se)	0·8	0·0	0·9	0·0	0·7	0·0	0·8	0·0	0·8	0·0	0·8	0·0
Alcohol, servings/d (se)	0·7	0·0	0·6	0·0	0·5	0·0	0·7	0·0	0·9	0·0	1·0	0·0
Fruit, servings/d (se)	1·0	0·0	1·0	0·0	1·1	0·0	1·0	0·0	1·0	0·0	0·9	0·0
Vegetable, servings/d (se)	1·6	0·0	1·5	0·0	1·5	0·0	1·6	0·0	1·7	0·0	1·7	0·0
Grain, servings/d (se)	6·5	0·0	6·7	0·0	6·1	0·1	6·4	0·1	6·5	0·1	6·6	0·1
Diary, servings/d (se)	1·6	0·0	1·6	0·0	1·3	0·0	1·5	0·0	1·6	0·0	1·8	0·0
Red meat, servings/d (se)	1·7	0·0	1·8	0·0	1·5	0·0	1·6	0·0	1·8	0·0	2·0	0·1
	*n*	%	*n*	%	*n*	%	*n*	%	*n*	%	*n*	%
Female, unweighted *n* (%, weighted)	21 710	51·2	9727	51·1	3421	60·8	4940	53·3	2186	48·6	1436	40·9
Ethnicity/race, unweighted *n* (%, weighted)												
White	19 404	68·4	7578	62·4	2031	57·0	4472	70·7	2790	81·3	2533	85·2
Black	9137	11·2	5695	16·8	1065	11·0	1542	7·9	553	4·8	282	3·2
Hispanic	10 681	13·4	4146	13·3	2145	22·0	2825	15·1	992	9·0	573	6·6
Other	3892	6·9	1868	7·6	626	9·9	840	6·3	333	4·9	225	5·0
Educational level, unweighted *n* (%, weighted)												
< High school	11 064	16·3	4678	16·3	1973	22·3	2604	16·2	1047	13·2	762	14·7
High school	10 097	24·1	4695	25·0	1249	22·7	2167	23·3	1032	22·2	954	25·1
> High school	21 953	59·6	9914	58·7	2645	55·0	4908	60·6	2589	64·6	1897	60·2
Smoking, unweighted *n* (%, weighted)												
Never	23 241	53·6	12 018	62·9	3407	58·4	4792	50·1	1943	42·5	1081	31·0
Former	10 737	24·9	3604	17·9	1516	24·7	2980	31·0	1510	32·9	1127	31·7
Now	9136	21·5	3665	19·1	944	16·9	1907	19·0	1215	24·6	1405	37·2
Physical activity^†^, unweighted *n* (%, weighted)												
Insufficient	32 567	73·0	14 374	72·8	4574	75·5	7361	72·7	3506	71·4	2752	73·9
Sufficient	10 547	27·0	4913	27·2	1293	24·5	2318	27·3	1162	28·6	861	26·1
Marital status, unweighted *n* (%, weighted)												
Married	25 735	62·2	10 658	57·2	3521	61·5	6189	66·8	3043	68·7	2324	66·5
Unmarried	7594	18·7	4870	26·6	702	14·5	1132	12·8	514	11·3	376	11·4
Other	978519·1		3759	16·2	1644	24·1	2358	20·4	1111	20·0	913	22·0
Family income^‡^, unweighted *n* (%, weighted)												
Low	12 252	20·5	6010	24·1	1797	24·0	2475	17·3	1062	14·0	908	16·5
Moderate	15 329	33·4	6798	34·1	2127	33·7	3532	32·6	1638	33·0	1234	32·4
High	12 399	40·0	5078	35·8	1447	34·9	2955	44·0	1664	47·8	1255	45·0
Missing value	3134	6·1	1401	6·0	496	7·4	717	6·1	304	5·2	216	6·0

Abbreviations: NHANES, National Health and Nutrition Examination Survey; se, standard error.

* Continuous data were expressed as weighted mean and se, while categorical variables were expressed by unweighted number of participants and weighted percentages.

† Sufficient physical activity was defined as ≥ 150 min of light to moderate intensity activity each week, or ≥ 75 min of vigorous-intensity activity, or an equivalent combination.

‡ Family income was calculated as the value of family income divided by official poverty threshold and categorized as low (≤ 1·30), moderate (1·31–3·50), and high (> 3·50).

### Coffee consumption and mortality risk

The median follow-up was 8·7 years with 6234 recorded deaths (1929 deaths caused by CVD and 1411 by cancer). Coffee consumption was significantly associated with reduction of all-cause and CVD mortality risks, but not with cancer mortality in all participants (Table 2 and eTable 4). We found a nonlinear association between coffee consumption and all-cause mortality (*P* = 0·01 for nonlinear trend, Table 2). Specifically, a U-shaped association was observed (*P* < 0·001 for nonlinearity), with approximately 2 cups of coffee daily yielding the greatest benefit in reducing the risk of mortality (see online supplementary material, Supplemental eFigure 3). Compared with non-coffee drinkers, those who consumed 1 to ≤ 2 cups per day had an 19 % lower risk of total mortality (HR, 0·81 (95 % CI, 0·75, 0·88)) and a 18 % lower risk of CVD mortality (HR, 0·82 (0·71, 0·94)). Higher coffee consumption (> 3 cups/day) was still associated with significant reduction of all-cause mortality (HR, 0·87 (0·78, 0·96)) and a 27 % lower risk of CVD mortality (HR, 0·73 (0·60, 0·90)). Similar association was observed in the sensitivity analyses (eTable 5–6).

**Table 2.** The association between coffee consumption and all-cause and cause-specific mortality^*^

Death cause	Metric	0 cup		≤ 1 cup		1 to ≤ 2 cups		2 to ≤ 3 cups		> 3 cups		*P* for linear trend	*P* for nonlinear trend
All participants													
All-cause	HR (95 % CI)	1 (reference)		0·93	0·84, 1·01	0·81	0·75, 0·88	0·89	0·81, 0·97	0·87	0·78, 0·96	0·001	0·01
	Case (total)	2225	19 287	1121	5867	1577	9679	741	4668	570	3613	–	–
CVD	HR (95 % CI)	1 (reference)		0·85·	0·72, 1	0·82	0·71, 0·94	0·83	0·69, 0·99	0·73	0·6, 0·9	<0·001	0·002
	Case (total)	705	19 287	354	5867	499	9679	221	4668	150	3613	–	–
Cancer	HR (95 % CI)	1 (reference)		0·92	0·75, 1·13	0·87	0·73, 1·03	1·1	0·91, 1·33	1·2	0·99, 1·46	0·17	0·07
	Case (total)	479	19 287	226	5867	356	9679	188	4668	162	3613	–	–
Other cause	HR (95 % CI)	1 (reference)		0·98	0·86, 1·11	0·79	0·7, 0·88	0·83	0·72, 0·95	0·8	0·69, 0·93	<0·001	0·002
	Case (total)	1041	19 287	541	5867	722	9679	332	4668	258	3613	–	–
Female													
All-cause	HR (95 % CI)	1 (reference)		0·92	0·81, 1·04	0·82	0·73, 0·92	0·82	0·71, 0·96	0·83	0·7, 0·98	0·006	0·02
	Case (total)	1012	9727	582	3421	675	4940	286	2186	174	1436	–	–
CVD	HR (95 % CI)	1 (reference)		0·87	0·69, 1·1	0·85	0·69, 1·05	0·79	0·59, 1·05	0·79	0·56, 1·11	0·06	0·09
	Case (total)	309	9727	185	3421	203	4940	76	2186	44	1436	–	–
Cancer	HR (95 % CI)	1 (reference)		0·96	0·72, 1·27	0·84	0·65, 1·08	0·96	0·71, 1·3	1·13	0·82, 1·57	0·94	0·7
	Case (total)	215	9727	102	3421	135	4940	74	2186	40	1436	–	–
Other cause	HR (95 % CI)	1 (reference)		0·93	0·78, 1·11	0·8	0·68, 0·94	0·8	0·65, 0·98	0·72	0·56, 0·92	0·004	0·007
	Case (total)	488	9727	295	3421	337	4940	136	2186	90	1436	–	–
Male													
All-cause	HR (95 % CI)	1 (reference)		0·94	0·82, 1·07	0·8	0·72, 0·9	0·94	0·83, 1·06	0·9	0·79, 1·02	0·11	0·25
	Case (total)	1213	9560	539	2446	902	4739	455	2482	396	2177	–	–
CVD	HR (95 % CI)	1 (reference)		0·84	0·66, 1·08	0·79	0·64, 0·96	0·86	0·68, 1·08	0·7	0·54, 0·9	0·01	0·01
	Case (total)	396	9560	169	2446	296	4739	145	2482	106	2177	–	–
Cancer	HR (95 % CI)	1 (reference)		0·89	0·67, 1·2	0·91	0·72, 1·15	1·2	0·94, 1·54	1·29·	1, 1·65	0·07	0·03
	Case (total)	264	9560	124	2446	221	4739	114	2482	122	2177	–	–
Other cause	HR (95 % CI)	1 (reference)		1·03	0·84, 1·25	0·77	0·65, 0·91	0·87	0·72, 1·05	0·87	0·72, 1·05	0·09	0·17
	Case (total)	553	9560	246	2446	385	4739	196	2482	168	2177	–	–

Abbreviations: CI, confidence interval; HR, hazard ratio; CVD, cardiovascular disease.

* Results adjusted for age, sex, race and ethnicity, educational attainment, marital status, family income, smoking, physical activity, consumption of alcohol, tea, fruit, vegetable, grain, protein food, and dairy. The *P*-value for the interaction of all-cause mortality between levels of coffee consumption and sexes was 0·70.

There was no significant interaction between coffee consumption level and sex (*P* = 0·70 for interaction), nor ethnicity (*P* = 0·56 for interaction), on all-cause mortality (Table 2). In line with the findings in the total cohort, a similar relationship between coffee consumption and risk of mortality were observed across different sexes (Table 2). Moderate coffee consumption (1 to ≤ 2 cups/day) was related to a lower risk of all-cause mortality in both male (HR, 0·80 (95 % CI, 0·72, 0·90)) and female (HR, 0·82 (0·73, 0·92)). There were significant disparities across ethnicities in the association between coffee consumption and the different causes of death (eTable 7). Specifically, moderate coffee consumption significantly reduced the risk of all-cause mortality in both White (HR, 0·81 (0·72, 0·91)) and Black (HR, 0·79 (0·66, 0·95)) populations, but not in Hispanic-Mexican group (HR, 0·87 (0·68, 1·10)); the same pattern held for cause-specific deaths. Similarly significant associations were observed for different coffee categories (eTable 8).

### Coffee consumption and life expectancy

**Figure 1.**
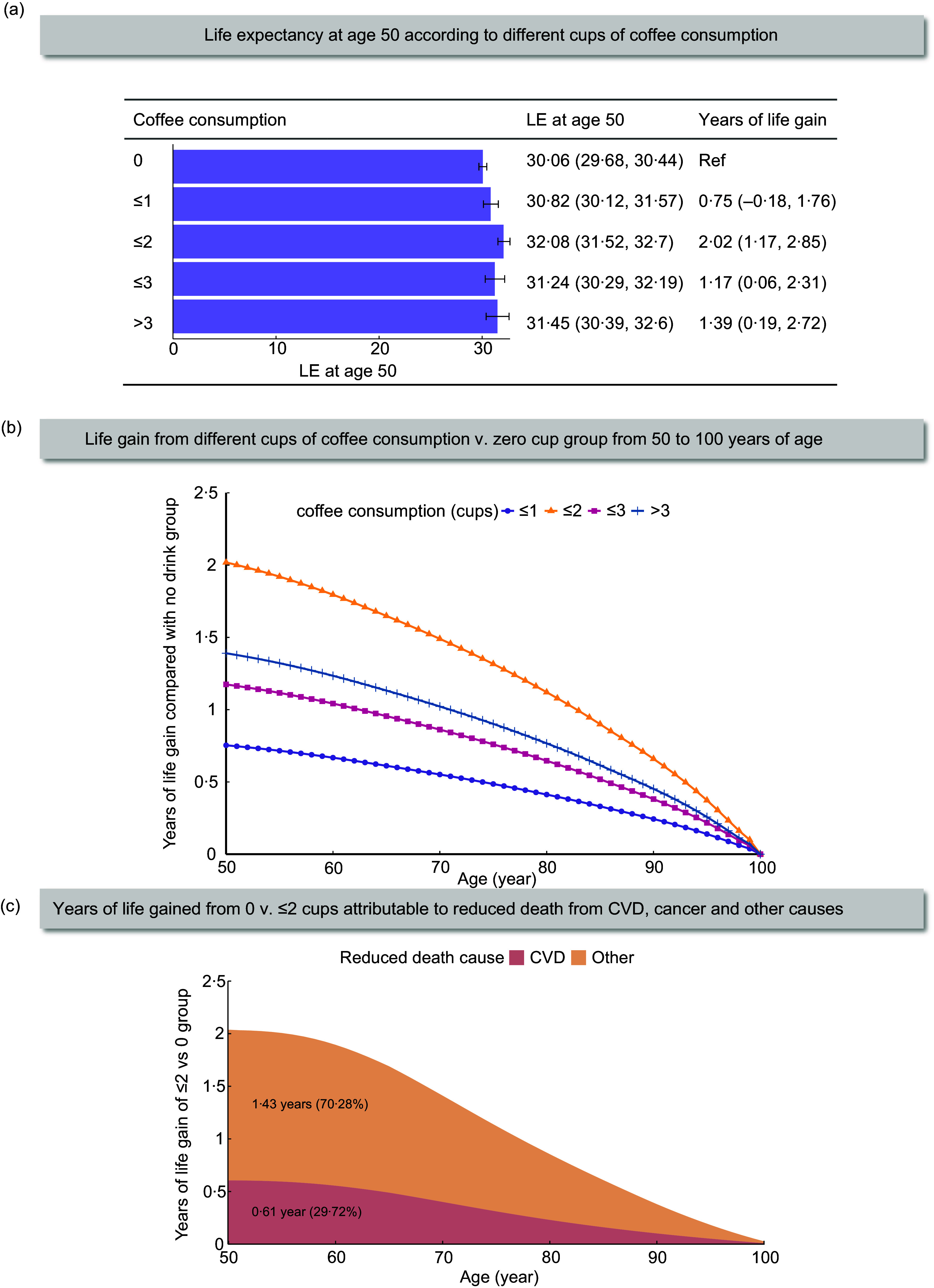
Estimates of cumulative survival time from 50 years of age onward among participants with different levels of coffee consumption in total cohort. LE = life expectancy; Ref = reference; CVD = cardiovascular disease. CI = confidence interval. The group of ≤ 2 represents 1 to ≤ 2 cups/day, and the group of ≤ 3 represents 2 to ≤ 3 cups/day. (a) Life expectancy at age 50 according to different coffee consumption levels. Error bars indicate 95 % CIs, which were estimated using Monte Carlo simulation with 1000 runs. (b) Life gain from different cups of coffee consumption from 50 to 100 years of age, compared with no coffee consumption. (c) Estimated years of life gained from 1 to ≤ 2 cups *v*. zero cup of coffee per day attributable to reduced death from CVD and other causes. Values in each area represent the gained years and proportion at age 50 attributed to corresponding causes.

At age 50 years, participants who consuming 0, ≤ 1, 1 to ≤ 2, 2 to ≤ 3, and > 3 cups of coffee per day had estimated LE of 30·06 years (95 % CI, 29·68, 30·44), 30·82 years (30·12, 31·57), 32·08 years (31·52, 32·70), 31·24 years (30·29, 32·19), and 31·45 years (30·39, 32·60), respectively (Figure 1). Consequently, compared with non-coffee drinkers, participants who consumed 1 to ≤ 2 cups of coffee per day could gain an average of 2·02 (1·17, 2·85) years in LE, with gains of 1·17 (0·06, 2·31) years for those consuming 2 to ≤ 3 cups/day and 1·39 (0·19, 2·72) years for those consuming > 3 cups/day. Consistent with the primary findings, all the sensitivity analyses revealed similar effects of coffee consumption on LE (eTable 6). Decomposition analysis revealed that life gain from moderate coffee consumption was mainly attributed to reduced CVD (Figure 1). Specifically, the gained LE from 1 to ≤ 2 cups/day of consumption is attributed to 0·61-year (29·72 %) reduction in CVD deaths.

### Coffee consumption and life expectancy by subgroups

**Figure 2.**
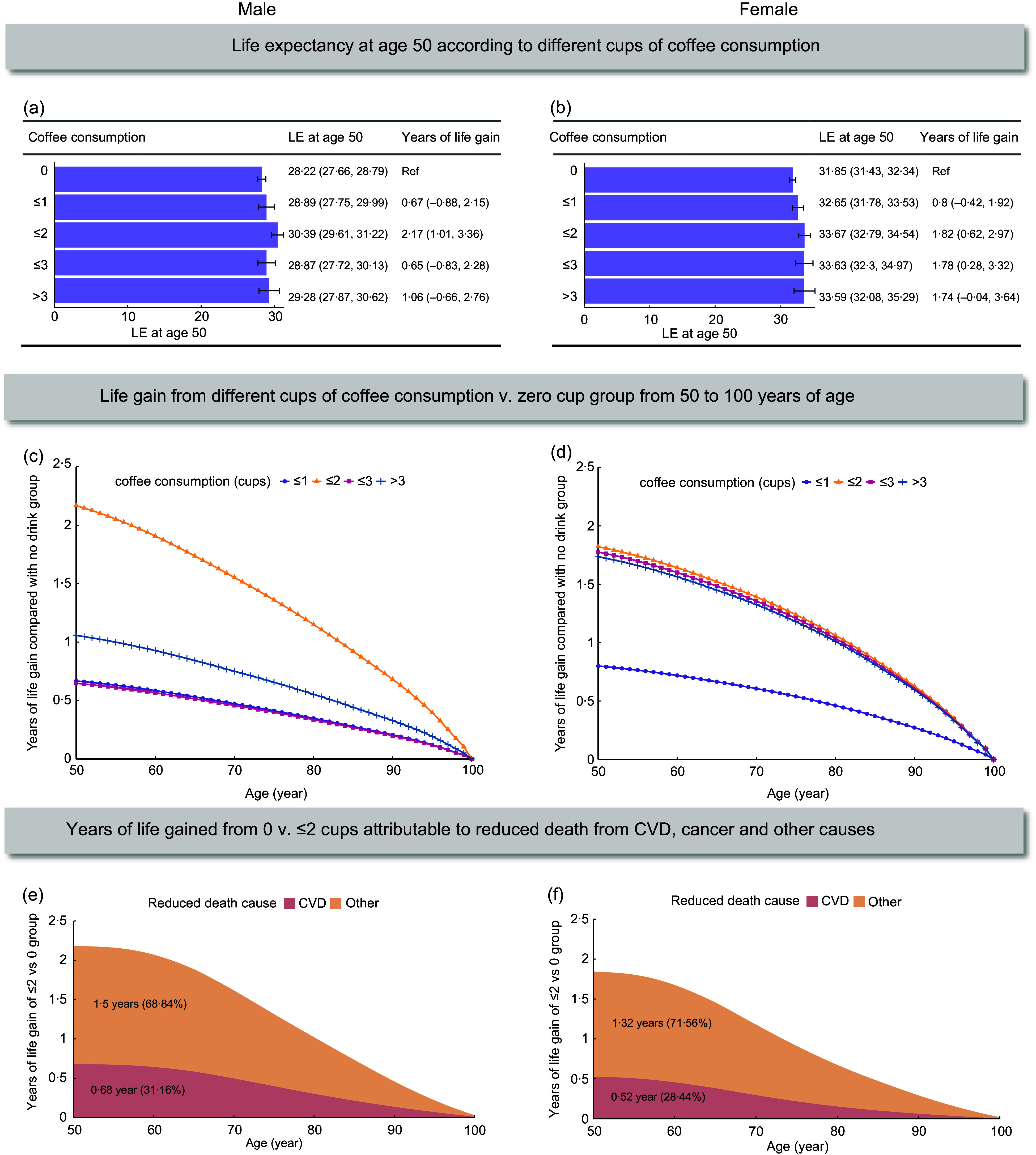
Estimates of cumulative survival time from 50 years of age onward among participants with different levels of coffee consumption by sex. LE = life expectancy; Ref = reference; CVD = cardiovascular disease. CI = confidence interval. The group of ≤ 2 represents 1 to ≤ 2 cups/day, and the group of ≤ 3 represents 2 to ≤ 3 cups/day. (a) (Male) and (b) (Female). Life expectancy at age 50 according to different coffee consumption levels. Error bars indicate 95 % CIs, which were estimated using Monte Carlo simulation with 1000 runs. (c) (Male) and (d) (Female). Life gain from different cups of coffee consumption from 50 to 100 years of age, compared with no coffee consumption. (e) (Male) and (f) (Female). Estimated years of life gained from 1 to ≤ 2 cups *v*. zero cup of coffee per day attributable to reduced death from CVD and other causes. Values in each area represent the gained years and proportion at age 50 attributed to corresponding causes.

The estimated LE of females was more than 3 years longer than that of males across different coffee consumption groups (Figure 2). Compared with non-consumers, moderate coffee consumption was rela ted to an increment of 2·17 (95 % CI, 1·01, 3·36) years in LE at age 50 for males and 1·82 (0·62, 2·97) years for females. On average, 31·16 % and 28·44 % of the gained LE at the age of 50 from moderate coffee consumption was attributable to reduced CVD for males and females, respectively. Interestingly, higher coffee consumption was still significantly associated with increased LE for females, but not for males.

**Figure 3.**
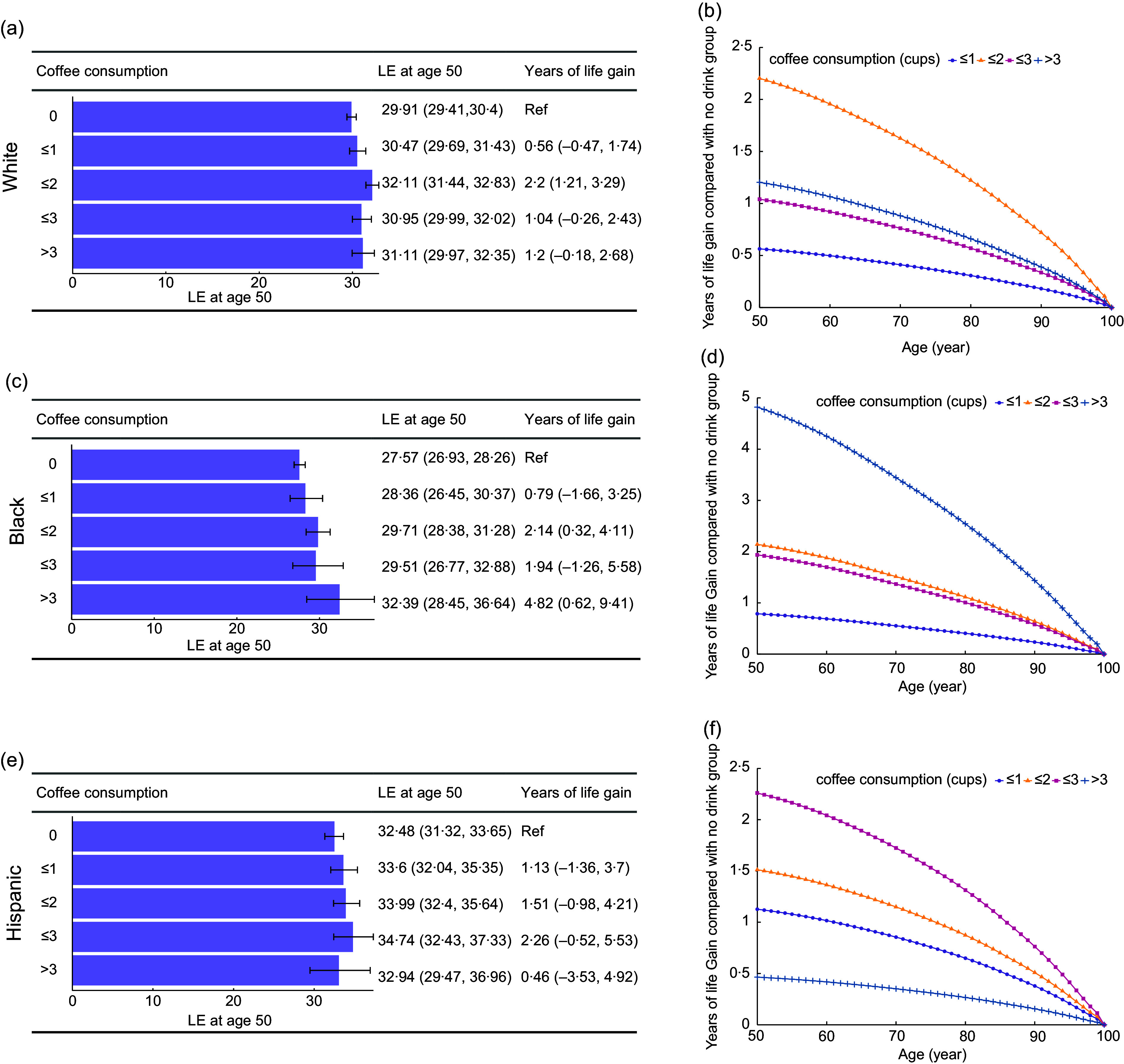
Estimates of cumulative survival time from 50 years of age onward among participants with different levels of coffee consumption by race and ethnicity. LE = life expectancy; Ref = reference; CVD = cardiovascular disease. CI = confidence interval. The group of ≤ 2 represents 1 to ≤ 2 cups/day, and the group of ≤ 3 represents 2 to ≤ 3 cups/day. (a) (White), (c) (Black) and (e) (Hispanic). Life expectancy at age 50 according to different coffee consumption levels. Error bars indicate 95 % CIs, which were estimated using Monte Carlo simulation with 1000 runs. (b) (White), (d) (Black) and (f) (Hispanic). Life gain from different cups of coffee consumption from 50 to 100 years of age, compared with no coffee consumption.

Both LE and life gain at the age of 50 were significantly different across ethnicities (Figure 3). Among different coffee consumption groups, we observed a longer estimated LE in the Hispanic population, followed by White and Black populations. Compared to non-coffee drinkers, those consuming 1 to ≤ 2 cups per day gained an estimated 2·20 (95 % CI, 1·21, 3·29) more years of LE for White population and 2·14 (0·32, 4·11) more years for Black population, while no significant LE benefit was observed in Hispanic individuals. Notably, Black participants who consumed > 3 cups of coffee per day gained 4·82 (0·62, 9·41) years compared to non-drinkers.

Across the various coffee categories, similar significant associations between coffee consumption and estimated LE were observed (see online supplementary material, Supplemental eFigure 4–6). Compared to non-coffee drinkers, individuals in the caffeinated group experienced the greatest LE gain when consuming 1 to ≤ 2 cups per day, with an increase of 2·22 (95 % CI, 1·39, 3·06) years, and 1·21 (0·34, 2·16) for ≤ 1 cup per day. Conversely, individuals in the decaffeinated group showed significant life gain when consuming > 3 cups per day, with an increase in 3·76 (0·70, 7·79) years (see online supplementary material, Supplemental eFigure 4). At age 50, moderate coffee consumption was associated with a gain in LE of 1·97 (1·20, 2·90) years in the group without sugar, whereas no significant associations were observed in the group with sugar across all levels of coffee consumption (see online supplementary material, Supplemental eFigure 5). Additionally, drinking 1 to ≤ 2 cups of non-instant coffee per day was associated with an expected LE gain of 2·04 (1·12, 2·96) years. For instant coffee, average gains of 1·73 (0·56, 2·98) years and 1·41 (0·09, 2·69) years in LE were observed in those who consumed ≤ 1 and 1 to ≤ 2 cups per day at the age of 50, respectively, while higher coffee consumption was not associated with increased LE (see online supplementary material, Supplemental eFigure 6).

## Discussion

Based on a nationally representative cohort, we found a significant positive association between coffee consumption and LE in the US adults. We estimated that moderate coffee consumption could prolong LE at age 50 by 2·02 years, with nearly one third of life gain attributed to reduced CVD deaths. Higher coffee consumption is still linked to increased LE, although the extent of this increase is not as pronounced as observed with moderate coffee consumption.

Numerous studies have shown that coffee consumption is associated with a reduced risk of mortality and various chronic diseases^(5–9,24–26)^. However, to the best of our knowledge, this is the first study examining the effect of coffee consumption on LE in US adults. Considering the long-term stagnation of LE in the US population, several studies have explored the correlation of combined lifestyle factors and LE in US adults^(12–16)^. Li et al. discovered that individuals who adhered to all five low-risk lifestyle factors experienced an increment in LE of 12·2 years for men and 14·0 years for women at the age of 50 compared to those with zero low-risk lifestyle factors^(12)^. Ma et al. estimated that participants with high cardiovascular health (evaluated by Life’s Essential 8 score) had 8·9 more years of LE compared to those with low cardiovascular health^(13)^. Compared to studies integrating multiple lifestyle factors, moderate coffee consumption may only modestly increase LE by 2 years at the age of 50 years, making it superficially less impactful. However, coffee is the most commonly consumed beverage in the US and even small individual benefits can have significant implications for the entire population. Our study found that the association between coffee consumption and life expectancy varied across different sexes and ethnicities, indicating a certain degree of heterogeneity. Specifically, females, Black individuals, and Hispanic individuals appeared to benefit more than males and White individuals. This suggests that genetic factors may play an important role and underscores the importance of implementing more targeted interventions in the future to enhance cost-effectiveness. Besides, compared to the complexity and intricacy of these multiple combined factors, coffee, as a modifiable protective lifestyle factor, serves as an excellent intervention and preventive tool for policymakers and the general public. It is not only easily implementable for policymakers but also readily understandable and acceptable to the public, as well as attractive to stakeholders.

Biologically, the protective effects of coffee are plausible due to the presence of various bioactive compounds. Caffeine is the most commonly available bioactive substance in coffee and has numerous health effects, such as promoting lipid metabolism, stimulating the central nervous system to enhance memory and cognitive function, and exerting antioxidant stress^(25,27–32)^. Polyphenols and other bioactive substances in coffee exhibit antioxidant and anti-inflammatory activities, reduce insulin resistance, and contribute to the prevention of various chronic diseases^(33–35)^. Notably, our estimates indicated that consuming approximately 2 cups of coffee per day could bring about the largest reduction in the risk of all-cause mortality, following a U-shaped pattern of association. Similar to our findings, an umbrella review reported that the consumption of 3 cups of coffee per day was associated with the largest reduction in the relative risk of all-cause mortality compared to no consumption^(5)^. Considering limited sample size, although our results showed a significant association with increased LE even with consumption of over 3 cups, it needs to be interpreted with caution. It should be mentioned that excessive coffee consumption could lead to a series of adverse effects caused by excessive caffeine intake, such as caffeine-dependence syndrome, anxiety, insomnia, headaches, and palpitations^(36–38)^. As our findings suggest, lower levels of coffee consumption in the caffeinated group were associated with increased life expectancy, while higher levels showed no association; conversely, the decaffeinated group showed the opposite. Therefore, for individuals, the potential benefits of drinking coffee should be balanced against the potential risks.

Interestingly, our analysis found that coffee consumption was associated with lower risk of all-cause and CVD mortality but not with cancer mortality. Reduction of CVD deaths contributed to nearly one third of life gain from moderate coffee consumption. The consensus among most studies is that coffee intake reduces the risk of CVD mortality^(5,39–42)^. A meta-analysis with 997 464 participants showed that consuming 3 cups of coffee per day could lower the risk of CVD mortality by 21 %, with the greatest benefit observed^(41)^. In line with these research findings, our analysis further strengthened the association, and we also found that a leading factor contributing to the extension of lifespan through tea consumption is attributed to reduced deaths from CVD. For benefit on cancer mortality, the controversy has persisted. Consistent with our findings, Alessio’s work showed that any level of coffee consumption was not associated with cancer mortality^(41)^. On the contrary, another meta-analysis observed that moderate coffee consumption (e.g. 2–4 cups/day) was associated with reduced cancer mortality, compared to non-drinkers^(40)^. Specifically, comprehensive umbrella review demonstrated that high *v*. low coffee consumption was associated with a lower risk of some specific cancers (such as prostate, endometrial, melanoma, and liver cancer), while no significant association was found with some others (such as gastric, colorectal, ovarian, thyroid, breast, pancreatic, oesophageal, or laryngeal cancers)^(5)^. There were harmful associations for coffee consumption with lung cancer and urinary tract cancer^(5)^. Moreover, a resent Mendelian randomisation study reported that coffee consumption was not associated with all-cancer risk but it was linked to a higher risk of digestive system cancers, particularly oesophageal cancer^(43)^. Therefore, if conditions permit, it would be better to explore the associations between the mortality of specific cancers and coffee consumption, rather than examining all-cancer mortality, and to assess whether coffee consumption contributes to the life gains attributed to the reduction in mortality from specific cancers.

Our study evaluated the association of coffee consumption on life expectancy using a nationally representative sample and examined various factors that may influence this relationship. The findings provide both a comprehensive and nuanced perspective, offering valuable insights for developing strategies to improve population life expectancy. This study had some limitations. First, one or two 24-hour dietary recall interviews were used to gather dietary data, which may not accurately reflect an individual’s daily dietary habits. Indeed, a single 24-hour dietary recall questionnaire is not well-suited for assessing long-term dietary intake. Therefore, we averaged the data from two dietary recall interviews to minimize bias, and the correlation coefficient of 0·74 indicates relatively high consistency. The undeniable fact is that food frequency questionnaires or repeated measurements of 24-hour dietary recall interviews can more accurately assess long-term dietary intake, which is expected to be conducted to validate or correct our findings in future research. Second, information on coffee consumption was collected by self-reporting, which may have introduced recall bias. To minimize recall biases, a standard set of measuring tools was used to help respondents report the volume and dimensions of consumed food items. Third, as an observational study, residual confounding remains despite adjusting for numerous potential confounding factors. Fourth, owing to the limited sample size, the estimates exhibited wide variations, especially in the subgroup analyses. Fifth, potential factors such as geographic area and climate, which may affect the study results, were not considered. Future research should pay more attention to these potential influences. Sixth, due to current limitations in statistical methodology, this study did not apply complex statistical models such as propensity score matching, mixed-effects models, competing risks models, or generalized additive models to produce more robust results. Future research should focus more on the advancement of statistical methods to achieve more stable and reliable findings. Seventh, factors such as coffee preparation method, roasting time, additives like milk, and concentration were not further analyzed due to a lack of relevant data, and we hope future studies can address these aspects effectively. Finally, the estimates were analyzed using dietary data at baseline; however, dietary patterns may change over time. These limitations should be addressed in future studies.

### Suggestions for future studies

In addition to the limitations discussed, there are several areas for improvement that future research could consider. From a study design perspective, increasing the frequency of dietary data collection and incorporating objective measures—such as food diaries or mobile app-based tracking—may help reduce recall bias and improve the accuracy of dietary assessments. From a data collection standpoint, gathering more detailed information related to coffee consumption, including coffee type, roast level, timing and frequency of intake, and drinking motivation, would enhance the comprehensiveness of the data. Collecting information on individual caffeine metabolism rates may also offer valuable insights. From an evidence-based perspective, future studies should consider the influence of other lifestyle factors and their potential interactions to produce more robust and high-quality evidence. Additionally, randomized controlled trials (RCTs) could be conducted to determine whether the observed relationship between coffee consumption and life expectancy is causal.

### Conclusion

Based on the nationally representative cohort, we firstly assessed the effect of coffee consumption on life expectancy and proved that coffee consumption, especially moderate coffee consumption (1–2 cups/day), was associated with increased life expectancy in general population, compared to non-coffee consumers. Our findings suggest that moderately consuming coffee could be a valuable part of a healthy diet and may be an adjustable effective intervention measure to increase life expectancy. Of course, none of these suggestions take into account the potential association on sensitive populations. Before implementing any intervention, it is essential to conduct precise screening to identify at-risk individuals and minimize the occurrence of adverse events and health harms.

## Supporting information

Yan et al. supplementary material Yan et al. supplementary material

## Data Availability

All data are publicly available and requests for data access should be made directly to the NHANES.

## Supplementary material

For supplementary material accompanying this paper visit https://doi.org/10.1017/S1368980025100888
