# Supplementary material for: Association between coffee consumption and life expectancy: a prospective cohort study from NHANES 2001–2018
Source: Public Health Nutr. 2025 Aug 22;28(1):e141. doi: 10.1017/S1368980025100888 (PMC12516614; doi:10.1017/S1368980025100888)
Supplement: Yan et al. supplementary material [file S1368980025100888sup001.pdf]

Supplementary Online Content

eTable 1. The food code of coffee along with the corresponding food description.

eTable 2. Characteristics of study participants in NHANES by different levels of coffee consumption in females.

eTable 3. Characteristics of study participants in NHANES by different levels of coffee consumption in males.

eTable 4. The Association between coffee consumption and cause-specific Mortality.

eTable 5. The association between coffee consumption and all-cause and cause specific mortality for sensitivity analysis by adjusting additional covariates.

eTable 6. The association between coffee consumption and all-cause mortality, life expectancy and life gain in four sensitivity analysis.

eTable 7. The association between coffee consumption and all-cause and cause-specific mortality by ethnicity.

eTable 8. The association between coffee consumption and all-cause and cause-specific mortality by different coffee categories.

eFigure 1. Flowchart diagram of enrolled participants.

eFigure 2. Observed and predicted mortality rates of US population in 2019.

eFigure 3. Restricted cubic spline fitting for the association between tea consumption with all-cause mortality.

eFigure 4. Estimates of cumulative survival time from 50 years of age onward among participants with different levels of coffee consumption by caffeine status.

eFigure 5. Estimates of cumulative survival time from 50 years of age onward among participants with different levels of coffee consumption by sugar addition status.

eFigure 6. Estimates of cumulative survival time from 50 years of age onward among participants with different levels of coffee consumption by preparation method.

eTable 1. The food code of coffee along with the corresponding food description.

Food code	Main description
92100000	coffee,
92100500	coffee, regular
92101000	coffee, made from ground, regular, brewed
92101500	coffee, made from ground, equal parts regular and decaffeinate, brewed, blend of regular and decaffeinated
92101600	coffee, turkish
92101610	coffee, espresso
92101630	coffee, espresso, decaffeinated
92101700	coffee, made from ground, regular, brewed, flavored
92101800	coffee, cuban
92101810	coffee, macchiato
92101820	coffee, macchiato, sweetened
92101850	coffee, cafe con leche
92101851	coffee, cafe con leche, decaffeinated
92101900	coffee, latte
92101901	coffee, latte, nonfat
92101903	coffee, latte, with non-dairy milk
92101904	coffee, latte, flavored
92101905	coffee, latte, nonfat, flavored
92101906	coffee, latte, with non-dairy milk, flavored
92101910	coffee, latte, decaffeinated
92101911	coffee, latte, decaffeinated, nonfat
92101917	coffee, latte, decaffeinated, flavored
92101918	coffee, latte, decaffeinated, nonfat, flavored
92101920	coffee, frappuccino, regular, blended coffee beverage, made with regular coffee, milk, and ice, sweetened
92101921	frozen coffee drink, nonfat
92101923	frozen coffee drink, with non-dairy milk
92101925	blended coffee beverage, made with regular coffee, milk, and ice, sweetened, with whipped cream
92101926	frozen coffee drink, nonfat, with whipped cream
92101930	coffee, frappuccino, blended coffee beverage, made with decaffeinated coffee, milk, and ice, sweetened
92101931	frozen coffee drink, decaffeinated, nonfat
92101935	blended coffee beverage, made with decaffeinated coffee, milk, and ice, sweetened, with whipped cream
92101936	frozen coffee drink, decaffeinated, nonfat, with whipped cream
92101950	coffee, cafe mocha
92101955	coffee, cafe mocha, nonfat
92101960	coffee, cafe mocha, with soy (non-dairy) milk

Food code	Main description
92101965	coffee, cafe mocha, decaffeinated
92101970	coffee, cafe mocha, decaffeinated, nonfat
92101975	coffee, cafe mocha, decaffeinated, with non-dairy milk
92102000	frozen mocha coffee drink
92102010	frozen mocha coffee drink, nonfat
92102020	frozen mocha coffee drink, with non-dairy milk
92102030	frozen mocha coffee drink, with whipped cream
92102040	frozen mocha coffee drink, nonfat, with whipped cream
92102050	frozen mocha coffee drink, with non-dairy milk and whipped cream
92102090	frozen mocha coffee drink, decaffeinated, with whipped cream
92102400	iced coffee, brewed
92102401	iced coffee, brewed, decaffeinated
92102450	iced coffee, pre-lightened and pre-sweetened
92102500	coffee, iced latte
92102501	coffee, iced latte, nonfat
92102502	coffee, iced latte, with non-dairy milk
92102503	coffee, iced latte, flavored
92102505	coffee, iced latte, with non-dairy milk, flavored
92102600	coffee, iced cafe mocha
92102602	coffee, iced cafe mocha, with non-dairy milk
92103000	coffee, regular, instant, reconstituted
92104000	coffee, instant, 50% less caffeine, reconstituted
92105000	coffee, liquid concentrate
92105010	coffee, made from liquid concentrate
92106000	coffee, acid neutralized, from powdered instant
92111000	coffee, decaffeinated
92111010	coffee, decaffeinated, made from ground, brewed
92114000	coffee, instant, decaffeinated, reconstituted
92121000	coffee, instant, pre-lightened and pre-sweetened with whitener and sugar, reconstituted
92121001	coffee, instant, decaffeinated, pre-lightened and pre-sweetened with sugar, reconstituted
92121010	coffee, instant, pre-sweetened with sugar, no whitener, reconstituted
92121020	coffee, mocha, instant, pre-lightened and pre-sweetened with sugar, with whitener, reconstituted
92121030	coffee, mocha, instant, pre-lightened and pre-sweetened with whitener and low-calorie sweetener, reconstituted
92121040	coffee, instant, pre-lightened and pre-sweetened with whitener and low-calorie sweetener, reconstituted
92121041	coffee, instant, decaffeinated, pre-lightened and pre-sweetened with low-calorie sweetener, reconstituted
92121050	coffee, mocha, instant, decaffeinated, with whitener, pre-lightened and pre-sweetened with low-calorie sweetener, reconstituted

Food code	Main description
92130000	coffee, regular, presweetened with sugar, pre-lightened
92130001	coffee, decaffeinated, presweetened with sugar, pre-lightened
92130005	coffee, regular, pre-sweetened with low-calorie sweetener, pre-lightened
92130006	coffee, decaffeinated, with pre-sweetened low-calorie sweetener, pre-lightened
92130010	coffee, pre-lightened, no sugar
92130011	coffee, decaffeinated, pre-lightened
92130020	coffee, presweetened with sugar
92130021	coffee, decaffeinated, pre-sweetened with sugar
92130030	coffee, pre-sweetened with low-calorie sweetener
92130031	coffee, decaffeinated, pre-sweetened with low-calorie sweetener
92152000	coffee and chicory, made from ground, brewed
92153100	coffee, decaffeinated, with cereal
92161000	coffee, cappuccino
92161001	coffee, cappuccino, nonfat
92161002	coffee, cappuccino, with non-dairy milk
92161005	cappuccino, sweetened
92162000	coffee, cappuccino, decaffeinated
92162001	coffee, cappuccino, decaffeinated, nonfat
92162005	cappuccino, decaffeinated, sweetened
92171000	coffee, bottled/canned
92171010	coffee, bottled/canned, light
92191000	coffee, dry instant powder,
92191100	coffee, dry instant powder, instant, not reconstituted
92191200	coffee, dry instant powder, instant, decaffeinated, not reconstituted
92191250	coffee, dry, acid neutralized
92191400	coffee, instant, pre-sweetened with sugar, not reconstituted
92192000	coffee, mocha, instant, dry instant powder with whitener, pre-lightened and pre-sweetened with sugar, not reconstituted
92192040	coffee, mocha, instant, decaffeinated, dry instant powder, with whitener, pre-lightened and pre-sweetened with low calorie sweetener, not reconstituted
92193000	coffee, instant, dry instant powder, with whitener, pre-lightened and pre-sweetened with sugar, not reconstituted
92193005	coffee, instant, decaffeinated, pre-lightened and pre-sweetened with sugar, not reconstituted
92193020	coffee, instant, dry instant powder, with whitener, pre-lightened and pre-sweetened with low calorie sweetener, not reconstituted

eTable 2. Characteristics of study participants in NHANES by different levels of coffee consumption in females*

Variable	Total	0 cup	≤1 cup	1 to ≤2 cups	2 to ≤3 cups	>3 cups
Age, y (SE)	48.3(0.2)	44.5(0.3)	52.7(0.5)	51.4(0.3)	51.0(0.4)	49.0(0.5)
Tea, servings/d (SE)	0.8(0.0)	0.9(0.0)	0.6(0.0)	0.7(0.0)	0.8(0.1)	0.7(0.1)
Alcohol, servings/d (SE)	0.4(0.0)	0.3(0.0)	0.3(0.0)	0.5(0.0)	0.5(0.0)	0.6(0.1)
Fruit, servings/d (SE)	1.0(0.0)	1.0(0.0)	1.0(0.0)	1.0(0.0)	0.9(0.0)	0.8(0.0)
Vegetable, servings/d (SE)	1.5(0.0)	1.4(0.0)	1.4(0.0)	1.5(0.0)	1.6(0.0)	1.5(0.0)
Grain, servings/d (SE)	5.5(0.0)	5.6(0.0)	5.4(0.1)	5.4(0.1)	5.6(0.1)	5.4(0.1)
Diary, servings/d (SE)	1.4(0.0)	1.4(0.0)	1.2(0.0)	1.3(0.0)	1.5(0.0)	1.5(0.0)
Red meat, servings/d (SE)	1.2(0.0)	1.3(0.0)	1.1(0.0)	1.2(0.0)	1.2(0.0)	1.3(0.1)
Ethnicity/race, unweighted N (%, weighted)						
White	9628(68.4)	3811(62.8)	1218(59.1)	2282(71.4)	1298(81.4)	1019(84.6)
Black	4664(11.8)	2886(17.5)	610(10.6)	818(8.6)	246(4.7)	104(3.1)
Hispanic	5475(12.9)	2091(12.3)	1266(20.9)	1429(14.1)	473(8.8)	216(6.3)
Other	1943(6.9)	939(7.4)	327(9.4)	411(6.0)	169(5.0)	97(6.0)
Educational level, unweighted N (%, weighted)						
< High school	5371(16.1)	2254(15.8)	1154(22.5)	1242(15.3)	451(12.9)	270(13.9)
High school	4919(23.4)	2209(23.6)	725(22.8)	1152(24.3)	476(22.2)	357(22.7)
> High school	11420(60.5)	5264(60.5)	1542(54.7)	2546(60.3)	1259(64.8)	809(63.3)
Smoking, unweighted N (%, weighted)						
Never	13698(60.3)	6832(68.5)	2346(65.9)	2919(57.2)	1099(49.1)	502(33.4)
Former	4159(20.6)	1432(15.4)	659(19.6)	1143(25.3)	554(26.8)	371(27.4)
Now	3853(19.1)	1463(16.1)	416(14.4)	878(17.5)	533(24.1)	563(39.2)
Physical activity†, unweighted N (%, weighted)						
Insufficient	17261(77.1)	7741(77.9)	2753(78.2)	3933(76.4)	1693(74.3)	1141(77.0)
Sufficient	4449(22.9)	1986(22.1)	668(21.8)	1007(23.6)	493(25.7)	295(23.0)
Marital status, unweighted N (%, weighted)						
Married	11685(59.1)	5040(56.3)	1791(57.1)	2782(62.4)	1279(64.8)	793(60.5)
Unmarried	3634(16.2)	2244(22.7)	403(12.7)	588(11.3)	237(9.7)	162(11.0)
Other	6391(24.6)	2443(21.1)	1227(30.2)	1570(26.2)	670(25.5)	481(28.6)
Family income‡, unweighted N (%, weighted)						
Low	6551(22.4)	3208(26.0)	1090(25.3)	1313(18.5)	540(15.9)	400(19.4)
Moderate	7675(33.9)	3392(34.1)	1227(33.6)	1802(33.6)	770(34.5)	484(33.6)
High	5888(37.6)	2441(33.8)	797(33.8)	1447(42.0)	730(44.4)	473(41.1)
Missing value	1596(6.1)	686(6.1)	307(7.4)	378(5.9)	146(5.2)	79(5.9)

Abbreviations: NHANES, National Health and Nutrition Examination Survey; SE, standard error.

*Continuous data were expressed as weighted mean and standard error, while categorical variables were expressed by unweighted number of participants and weighted percentages.

†Sufficient physical activity was defined as ≥150 minutes of light to moderate intensity activity each week, or ≥75 minutes of vigorous-intensity activity, or an equivalent combination.

‡Family income was calculated as the value of family income divided by official poverty threshold and categorized as low (≤1.30), moderate (1.31–3.50), and high (>3.50).

eTable 3. Characteristics of study participants in NHANES by different levels of coffee consumption in males*

Variable	Total	0 cup	≤1 cup	1 to ≤2 cups	2 to ≤3 cups	>3 cups
Age, y (SE)	46.4(0.2)	41.3(0.3)	50.3(0.6)	50.4(0.4)	51.0(0.4)	50.0(0.4)
Tea, servings/d (SE)	0.9(0.0)	1.0(0.0)	0.7(0.1)	0.8(0.0)	0.9(0.1)	0.9(0.1)
Alcohol, servings/d (SE)	1.0(0.0)	0.9(0.0)	0.8(0.1)	1.0(0.1)	1.2(0.1)	1.2(0.1)
Fruit, servings/d (SE)	1.0(0.0)	1.1(0.0)	1.1(0.1)	1.1(0.0)	1.0(0.0)	0.9(0.0)
Vegetable, servings/d (SE)	1.7(0.0)	1.7(0.0)	1.6(0.0)	1.7(0.0)	1.8(0.0)	1.7(0.0)
Grain, servings/d (SE)	7.6(0.0)	7.8(0.1)	7.3(0.1)	7.4(0.1)	7.3(0.1)	7.5(0.1)
Diary, servings/d (SE)	1.8(0.0)	1.9(0.0)	1.5(0.0)	1.6(0.0)	1.8(0.0)	2.0(0.0)
Red meat, servings/d (SE)	2.2(0.0)	2.2(0.0)	2.0(0.1)	2.1(0.1)	2.3(0.1)	2.4(0.1)
Ethnicity/race, unweighted N (%, weighted)						
White	9776(68.5)	3767(61.9)	813(53.9)	2190(70.0)	1492(81.2)	1514(85.6)
Black	4473(10.6)	2809(16.0)	455(11.7)	724(7.1)	307(4.9)	178(3.3)
Hispanic	5206(13.9)	2055(14.4)	879(23.8)	1396(16.3)	519(9.2)	357(6.9)
Other	1949(6.9)	929(7.7)	299(10.7)	429(6.7)	164(4.7)	128(4.2)
Educational level, unweighted N (%, weighted)						
< High school	5693(16.6)	2424(16.7)	819(21.9)	1362(17.1)	596(13.4)	492(15.2)
High school	5178(24.7)	2486(26.5)	524(22.6)	1015(22.1)	556(22.2)	597(26.8)
> High school	10533(58.7)	4650(56.8)	1103(55.5)	2362(60.8)	1330(64.4)	1088(58.0)
Smoking, unweighted N (%, weighted)						
Never	9543(46.6)	5186(57.1)	1061(46.7)	1873(41.9)	844(36.3)	579(29.4)
Former	6578(29.4)	2172(20.6)	857(32.5)	1837(37.4)	956(38.6)	756(34.7)
Now	5283(24.0)	2202(22.4)	528(20.8)	1029(20.6)	682(25.1)	842(35.9)
Physical activity†, unweighted N (%, weighted)						
Insufficient	15306(68.8)	6633(67.5)	1821(71.2)	3428(68.5)	1813(68.6)	1611(71.8)
Sufficient	6098(31.2)	2927(32.5)	625(28.8)	1311(31.5)	669(31.4)	566(28.2)
Marital status, unweighted N (%, weighted)						
Married	14050(65.5)	5618(58.2)	1730(68.3)	3407(71.9)	1764(72.4)	1531(70.7)
Unmarried	3960(21.2)	2626(30.7)	299(17.2)	544(14.4)	277(12.8)	214(11.8)
Other	3394(13.3)	1316(11.1)	417(14.5)	788(13.8)	441(14.8)	432(17.5)
Family income‡, unweighted N (%, weighted)						
Low	5701(18.5)	2802(22.1)	707(22.1)	1162(15.9)	522(12.3)	508(14.6)
Moderate	7654(322.9)	3406(34.1)	900(33.8)	1730(31.5)	868(31.5)	750(31.6)
High	6511(42.5)	2637(37.8)	650(36.7)	1508(46.2)	934(51.1)	782(47.7)
Missing value	1538(6.1)	715(5.9)	189(7.4)	339(6.4)	158(5.2)	137(6.1)

Abbreviations: NHANES, National Health and Nutrition Examination Survey; SE, standard error.

*Continuous data were expressed as weighted mean and standard error, while categorical variables were expressed by unweighted number of participants and weighted percentages.

†Sufficient physical activity was defined as ≥150 minutes of light to moderate intensity activity each week, or ≥75 minutes of vigorous-intensity activity, or an equivalent combination.

‡Family income was calculated as the value of family income divided by official poverty threshold and categorized as low (≤1.30), moderate (1.31–3.50), and high (>3.50).

eTable 4. The Association between coffee consumption and cause-specific Mortality*

Death cause	Metric	0 cup	≤1 cup	1 to ≤2 cups	2 to ≤3 cups	>3 cups
Cancer	HR (95% CI)	1 (reference)	0.92 (0.75, 1.13)	0.87 (0.73, 1.03)	1.1 (0.91, 1.33)	1.2 (0.99, 1.46)
	Case(total)	479(19287)	226(5867)	356(9679)	188(4668)	162(3613)
Heart disease	HR (95% CI)	1 (reference)	0.86 (0.72, 1.04)	0.85 (0.72, 0.99)	0.88 (0.72, 1.06)	0.78 (0.63, 0.97)
	Case(total)	579(19287)	283(5867)	419(9679)	184(4668)	129(3613)
Stroke	HR (95% CI)	1 (reference)	0.79 (0.53, 1.17)	0.68 (0.48, 0.98)	0.62 (0.38, 0.99)	0.51 (0.28, 0.91)
	Case(total)	126(19287)	71(5867)	80(9679)	37(4668)	21(3613)
Respiratory	HR (95% CI)	1 (reference)	1.05 (0.7, 1.59)	1.09 (0.78, 1.54)	1.08 (0.73, 1.62)	1.48 (1.01, 2.16)
	Case(total)	91(19287)	54(5867)	98(9679)	43(4668)	54(3613)
Neurodegenerative	HR (95% CI)	1 (reference)	1.3 (0.83, 2.03)	1.07 (0.71, 1.61)	0.96 (0.56, 1.63)	0.83 (0.42, 1.63)
	Case(total)	70(19287)	52(5867)	65(9679)	24(4668)	12(3613)
Diabetes	HR (95% CI)	1 (reference)	0.85 (0.52, 1.37)	0.58 (0.37, 0.91)	0.71 (0.41, 1.2)	1.08 (0.66, 1.75)
	Case(total)	78(19287)	41(5867)	50(9679)	29(4668)	18(3613)
Infection	HR (95% CI)	1 (reference)	1.17 (0.59, 2.32)	0.82 (0.42, 1.59)	1.15 (0.54, 2.42)	1.52 (0.7, 3.28)
	Case(total)	40(19287)	27(5867)	29(9679)	13(4668)	13(3613)
Kidney disease	HR (95% CI)	1 (reference)	0.41 (0.19, 0.86)	0.69 (0.41, 1.18)	0.62 (0.31, 1.27)	1.02 (0.53, 1.96)
	Case(total)	63(19287)	20(5867)	35(9679)	16(4668)	14(3613)
Other	HR (95% CI)	1 (reference)	1 (0.85, 1.19)	0.76 (0.65, 0.88)	0.85 (0.7, 1.02)	0.68 (0.55, 0.84)
	Case(total)	614(19287)	325(5867)	408(9679)	186(4668)	126(3613)

Abbreviations: CI indicates confidence interval; HR, hazard ratio.
*Results adjusted for age, sex, race and ethnicity, educational attainment, marital status, family income, smoking, physical activity, consumption of alcohol, tea, fruit, vegetable, grain, protein food, and dairy.

eTable 5. The association between coffee consumption and all-cause and cause specific mortality for sensitivity analysis by adjusting additional covariates.

Death cause	0 cup	≤1 cup	≤2 cups	≤3 cups	>3 cups
Adjusting physical conditions*					
All cause	1 (reference)	0.93 (0.84, 1.01)	0.81 (0.75, 0.88)	0.89 (0.81, 0.97)	0.87 (0.78, 0.96)
CVD	1 (reference)	0.85 (0.72, 1)	0.82 (0.71, 0.94)	0.83 (0.69, 0.99)	0.73 (0.6, 0.9)
Cancer	1 (reference)	0.92 (0.75, 1.13)	0.87 (0.73, 1.03)	1.1 (0.91, 1.33)	1.2 (0.99, 1.46)
Other	1 (reference)	0.98 (0.86, 1.11)	0.79 (0.7, 0.88)	0.83 (0.72, 0.95)	0.8 (0.69, 0.93)
Adjusting healthy diet and physical conditions**					
All cause	1 (reference)	0.91 (0.83, 0.99)	0.79 (0.73, 0.86)	0.84 (0.76, 0.92)	0.88 (0.79, 0.98)
CVD	1 (reference)	0.82 (0.7, 0.97)	0.81 (0.7, 0.93)	0.81 (0.67, 0.97)	0.74 (0.6, 0.91)
Cancer	1 (reference)	0.94 (0.77, 1.15)	0.85 (0.72, 1.01)	1.06 (0.87, 1.28)	1.24 (1.01, 1.51)
Other	1 (reference)	0.94 (0.83, 1.07)	0.76 (0.68, 0.85)	0.76 (0.66, 0.88)	0.8 (0.69, 0.94)

Abbreviations: CVD, cardiovascular diseases
All results were expressed as hazard ratio and 95% confidence interval.
*Results adjusted for age, sex, race and ethnicity, educational attainment, marital status, family income, smoking, physical activity, consumption of alcohol, tea, fruit, vegetable, grain, protein food, and dairy, diabetes, hypertension and dyslipidemia.
**Results adjusted for age, sex, race and ethnicity, educational attainment, marital status, family income, smoking, physical activity, healthy plant-based diet index, diabetes, hypertension and dyslipidemia.

Definition of Diabetes, Hypertension and Dyslipidemia

Diabetes was defined as follows: diagnosed by a health care professional, anti-diabetes medication use, hemoglobin A1c ≥ 6.5%, fasting PG ≥ 7.0 mmol/L with no caloric intake for at least 8h. Hypertension was defined as the confirmation of diagnosis by a doctor or health professional, or the use of antihypertensive medication, or systolic BP values ≥ 140 mmHg or diastolic BP values ≥ 90 mmHg. Dyslipidemia was defined as fasting TC ≥ 240 mg/dL, fasting triglyceride ≥ 240 mg/dL, fasting LDL-C ≥ 160 mg/dL, HDL-C < 40 mg/dL, the use of lipid-lowering medication or self-reported history of dyslipidemia.

Definition of Healthy Plant-Based diet index

The HPDI was developed using a methodology akin to that of AMED. Initially, we categorized 18 food groups based on their nutritional profiles and culinary characteristics . These groups were classified into three main categories: healthy plant foods (including whole grains, fruits, vegetables, nuts, legumes, vegetable oils, tea, and coffee), less healthy plant foods (such as fruit juices, refined grains, potatoes, sugar-sweetened beverages, sweets, and desserts), and animal foods (including animal fats like butter or lard, dairy, eggs, fish and seafood, meat, and other animal-based items). Participants' intake of these 18 food groups was evaluated in quintiles, with each quintile assigned scores ranging from one to five. Similar to AMED, positive scores were assigned to healthy plant food groups, while less healthy plant and animal food groups received reversed scores. The HPDI score, calculated by summing these scores across all food groups, ranged from 18 to 90, with higher scores indicating greater adherence to a healthier plant-focused diet.

Reference: Satija A, Bhupathiraju SN, Spiegelman D, et al. Healthful and Unhealthful Plant-Based Diets and the Risk of Coronary Heart Disease in U.S. Adults. J Am Coll Cardiol. 2017. 70(4): 411-422.

Healthful Plant-based Diet Index Components and Criteria for Scoring

Component	Criteria for minimum score of 1	Criteria for maximum score of 5
Healthy Plant Food Groups, serving/d		
Whole grains	Lowest quintile	Highest quintile
Fruits	Lowest quintile	Highest quintile
Vegetables	Lowest quintile	Highest quintile
Nuts	Lowest quintile	Highest quintile
Legumes	Lowest quintile	Highest quintile
Vegetable oils	Lowest quintile	Highest quintile
Tea & Coffee	Lowest quintile	Highest quintile
Unhealthy Plant Food Groups, serving/d		
Fruit juices	Highest quintile	Lowest quintile
Refined grains	Highest quintile	Lowest quintile
Potatoes	Highest quintile	Lowest quintile
Sugar sweetened beverages	Highest quintile	Lowest quintile
Sweets and desserts	Highest quintile	Lowest quintile
Animal Food Groups, serving/d		
Animal fat	Highest quintile	Lowest quintile
Dairy	Highest quintile	Lowest quintile
Egg	Highest quintile	Lowest quintile
Fish or seafood	Highest quintile	Lowest quintile
Meat	Highest quintile	Lowest quintile
Miscellaneous animal-based foods	Highest quintile	Lowest quintile
Total	18	90

eTable 6. The association between coffee consumption and all-cause mortality, life expectancy and life gain in four sensitivity analysis*

Index	0 cup	≤1 cup	1 to ≤2 cups	2 to ≤3 cups	>3 cups
Excluding those who don't drink coffee					
HR	NA	1 (reference)	0.88 (0.79,0.97)	0.96 (0.86,1.08)	0.95 (0.84,1.07)
LE	NA	30.3(29.77, 30.84)	31.56(31, 32.17)	30.66(29.75, 31.56)	30.72(29.69, 31.89)
Life gain	NA	reference	1.27(0.3, 2.33)	0.38(-0.9, 1.58)	0.43(-0.93, 1.77)
Excluding participants with missing covariates					
HR	1 (reference)	0.9 (0.82,1)	0.82 (0.76,0.89)	0.88 (0.8,0.97)	0.91 (0.82,1.01)
LE	28.19(27.78, 28.62)	29.18(28.48, 29.91)	30.17(29.55, 30.86)	29.39(28.43, 30.46)	29.08(27.96, 30.27)
Life gain	reference	0.98(-0.02, 1.99)	1.97(1.04, 2.95)	1.22(0.05, 2.48)	0.9(-0.45, 2.26)
Imputing missing covariate data with multiple imputation					
HR	1 (reference)	0.92 (0.84,1.01)	0.81 (0.75,0.88)	0.88 (0.8,0.97)	0.87 (0.78,0.96)
LE	30.06(29.69, 30.44)	30.82(30.13, 31.57)	32.07(31.53, 32.68)	31.26(30.31, 32.2)	31.48(30.39, 32.63)
Life gain	reference	0.77(-0.18, 1.76)	1.99(1.14, 2.85)	1.19(0.08, 2.33)	1.41(0.2, 2.74)
Excluding those who died within two years					
HR	1 (reference)	0.92 (0.83,1.02)	0.82 (0.75,0.9)	0.91 (0.82,1.01)	0.91 (0.82,1.02)
LE	28.26(27.82, 28.68)	29.05(28.29, 29.84)	30.19(29.58, 30.88)	29.14(28.14, 30.06)	29.22(28.15, 30.38)
Life gain	reference	0.8(-0.22, 1.86)	1.94(1.01, 2.95)	0.89(-0.37, 2.1)	0.95(-0.34, 2.28)

Abbreviations: HR, hazard ratio; LE, life expectancy.

*Results adjusted for age, sex, race and ethnicity, educational attainment, marital status, family income, smoking, physical activity, consumption of alcohol, tea, fruit, vegetable, grain, protein food, and dairy. All results were expressed as estimation and 95% CI.

eTable 7. The association between coffee consumption and all-cause and cause-specific mortality by ethnicity*

Death cause	Metric	0 cup	≤1 cup	1 to ≤2 cups	2 to ≤3 cups	>3 cups	P for linear trend	P for nonlinear trend
White								
All-cause	HR (95% CI)	1 (reference)	0.94 (0.83, 1.08)	0.8 (0.71, 0.89)	0.9 (0.79, 1.02)	0.88 (0.77, 1.01)	0.02	0.07
	Case(total)	1199(7578)	645(2031)	1016(4472)	551(2790)	459(2533)	-	-
CVD	HR (95% CI)	1 (reference)	0.88 (0.69, 1.12)	0.8 (0.65, 0.98)	0.83 (0.65, 1.06)	0.79 (0.6, 1.03)	0.01	0.03
	Case(total)	387(7578)	213(2031)	335(4472)	167(2790)	125(2533)	-	-
Cancer	HR (95% CI)	1 (reference)	0.92 (0.68, 1.24)	0.9 (0.71, 1.14)	1.14 (0.88, 1.48)	1.21 (0.93, 1.58)	0.13	0.08
	Case(total)	238(7578)	117(2031)	223(4472)	132(2790)	120(2533)	-	-
Other cause	HR (95% CI)	1 (reference)	1 (0.85, 1.17)	0.76 (0.64, 0.90)	0.84 (0.7, 1.01)	0.8 (0.66, 0.98)	0.003	0.009
	Case(total)	574(7578)	315(2031)	458(4472)	252(2790)	214(2533)	-	-
Black								
All-cause	HR (95% CI)	1 (reference)	0.93 (0.78, 1.11)	0.82 (0.69, 0.98)	0.84 (0.65, 1.08)	0.64 (0.44, 0.92)	0.005	0.01
	Case(total)	666(5695)	215(1065)	240(1542)	77(553)	43(282)	-	-
CVD	HR (95% CI)	1 (reference)	0.73 (0.52, 1.03)	0.73 (0.53, 1)	0.78 (0.48, 1.26)	0.34 (0.14, 0.82)	0.02	0.02
	Case(total)	213(5695)	68(1065)	74(1542)	19(553)	10(282)	-	-
Cancer	HR (95% CI)	1 (reference)	1.04 (0.72, 1.5)	0.9 (0.64, 1.27)	0.91 (0.55, 1.5)	1.33 (0.76, 2.32)	0.93	0.81
	Case(total)	159(5695)	52(1065)	62(1542)	26(553)	19(282)	-	-
Other cause	HR (95% CI)	1 (reference)	1.02 (0.79, 1.33)	0.84 (0.65, 1.09)	0.84 (0.57, 1.24)	0.48 (0.26, 0.91)	0.05	0.04
	Case(total)	294(5695)	95(1065)	104(1542)	32(553)	14(282)	-	-
Hispanic								
All-cause	HR (95% CI)	1 (reference)	0.89 (0.71, 1.12)	0.85 (0.68, 1.06)	0.78 (0.57, 1.08)	0.95 (0.66, 1.37)	0.26	0.41
	Case(total)	269(4146)	232(2145)	268(2825)	97(992)	55(573)	-	-
CVD	HR (95% CI)	1 (reference)	0.81 (0.51, 1.27)	0.94 (0.63, 1.42)	0.6 (0.31, 1.17)	0.98 (0.5, 1.91)	0.6	0.66
	Case(total)	76(4146)	64(2145)	78(2825)	27(992)	15(573)	-	-
Cancer	HR (95% CI)	1 (reference)	1.03 (0.64, 1.67)	0.81 (0.5, 1.31)	1.35 (0.75, 2.42)	1.07 (0.5, 2.26)	0.75	0.61
	Case(total)	62(4146)	52(2145)	61(2825)	26(992)	17(573)	-	-
Other cause	HR (95% CI)	1 (reference)	0.87 (0.63, 1.2)	0.83 (0.61, 1.13)	0.67 (0.42, 1.08)	0.88 (0.52, 1.5)	0.34	0.42
	Case(total)	131(4146)	116(2145)	129(2825)	44(992)	23(573)	-	-

Abbreviations: CI indicates confidence interval; HR, hazard ratio; CVD, cardiovascular disease.

*Results adjusted for age, sex, race and ethnicity, educational attainment, marital status, family income, smoking, physical activity, consumption of alcohol, tea, fruit, vegetable, grain, protein food, and dairy. The p-value for the interaction of all-cause mortality between levels of coffee consumption and ethnicities was 0.56.

eTable 8. The association between coffee consumption and all-cause and cause-specific mortality by different coffee categories*

Subgroup	Death cause	0 cup	≤1 cup	1 to ≤2 cups	2 to ≤3 cups	>3 cups	P for linear trend	P for nonlinear trend
With sugar	All-cause	1 (reference)	0.83 (0.52, 1.3)	0.7 (0.48, 1.02)	0.85 (0.57, 1.27)	0.58 (0.39, 0.88)	0.04	0.05
	CVD	1 (reference)	0.63 (0.26, 1.54)	0.77 (0.39, 1.52)	0.74 (0.33, 1.67)	0.18 (0.04, 0.82)	0.002	0.002
	Cancer	1 (reference)	1.1 (0.46, 2.67)	0.4 (0.14, 1.16)	0.88 (0.37, 2.09)	1.07 (0.56, 2.06)	0.75	0.85
	Other cause	1 (reference)	0.84 (0.44, 1.62)	0.79 (0.48, 1.29)	0.9 (0.52, 1.54)	0.56 (0.32, 1)	0.17	0.19
Without sugar	All-cause	1 (reference)	0.93 (0.85, 1.02)	0.82 (0.75, 0.88)	0.89 (0.81, 0.98)	0.89 (0.8, 0.98)	0.003	0.02
	CVD	1 (reference)	0.86 (0.72, 1.01)	0.82 (0.71, 0.94)	0.83 (0.69, 0.99)	0.76 (0.62, 0.93)	0.002	0.005
	Cancer	1 (reference)	0.91 (0.74, 1.12)	0.89 (0.75, 1.05)	1.11 (0.92, 1.35)	1.21 (0.99, 1.48)	0.14	0.06
	Other cause	1 (reference)	0.98 (0.86, 1.12)	0.79 (0.7, 0.89)	0.83 (0.72, 0.95)	0.82 (0.7, 0.95)	0.001	0.003
Instant coffee	All-cause	1 (reference)	0.83 (0.73, 0.95)	0.86 (0.76, 0.98)	0.91 (0.73, 1.13)	0.84 (0.6, 1.17)	0.003	0.007
	CVD	1 (reference)	0.82 (0.65, 1.03)	0.76 (0.6, 0.96)	0.79 (0.51, 1.21)	0.66 (0.33, 1.33)	<0.001	<0.001
	Cancer	1 (reference)	0.74 (0.54, 1.01)	0.93 (0.71, 1.21)	1.22 (0.8, 1.85)	1.28 (0.71, 2.3)	0.85	0.62
	Other cause	1 (reference)	0.88 (0.73, 1.05)	0.91 (0.76, 1.08)	0.85 (0.62, 1.18)	0.76 (0.46, 1.25)	0.02	0.01
Non-instant coffee	All-cause	1 (reference)	0.94 (0.85, 1.04)	0.81 (0.74, 0.88)	0.9 (0.81, 1)	0.88 (0.79, 0.98)	0.003	0.02
	CVD	1 (reference)	0.86 (0.72, 1.04)	0.82 (0.7, 0.96)	0.84 (0.69, 1.02)	0.74 (0.59, 0.92)	0.002	0.003
	Cancer	1 (reference)	0.97 (0.78, 1.21)	0.96 (0.8, 1.14)	1.1 (0.89, 1.35)	1.23 (0.99, 1.51)	0.1	0.05
	Other cause	1 (reference)	0.98 (0.84, 1.13)	0.74 (0.65, 0.84)	0.86 (0.74, 1)	0.82 (0.69, 0.96)	<0.001	0.003
Caffeinated	All-cause	1 (reference)	0.88 (0.8, 0.97)	0.8 (0.73, 0.86)	0.9 (0.81, 0.99)	0.89 (0.8, 1)	0.001	0.008
	CVD	1 (reference)	0.84 (0.71, 1)	0.79 (0.68, 0.92)	0.82 (0.68, 0.99)	0.75 (0.6, 0.93)	<0.001	<0.001
	Cancer	1 (reference)	0.93 (0.76, 1.14)	0.88 (0.74, 1.05)	1.2 (0.99, 1.45)	1.22 (0.99, 1.51)	0.15	0.9
	Other cause	1 (reference)	0.89 (0.78, 1.02)	0.77 (0.68, 0.86)	0.81 (0.7, 0.94)	0.83 (0.71, 0.98)	<0.001	0.004
Decaffeinated	All-cause	1 (reference)	0.88 (0.74, 1.03)	0.96 (0.83, 1.11)	0.88 (0.69, 1.13)	0.67 (0.48, 0.93)	0.03	0.03
	CVD	1 (reference)	0.64 (0.46, 0.88)	0.91 (0.7, 1.19)	0.85 (0.54, 1.36)	0.72 (0.4, 1.29)	0.11	0.16
	Cancer	1 (reference)	1.15 (0.82, 1.61)	0.99 (0.71, 1.36)	0.55 (0.28, 1.05)	1.09 (0.63, 1.88)	0.98	0.83
	Other cause	1 (reference)	0.93 (0.74, 1.17)	0.98 (0.79, 1.21)	1.06 (0.76, 1.48)	0.44 (0.24, 0.79)	0.02	0.006

Abbreviations: CI indicates confidence interval; HR, hazard ratio; CVD, cardiovascular disease.

*Results adjusted for age, sex, race and ethnicity, educational attainment, marital status, family income, smoking, physical activity, consumption of alcohol, tea, fruit, vegetable, grain, protein food, and dairy.

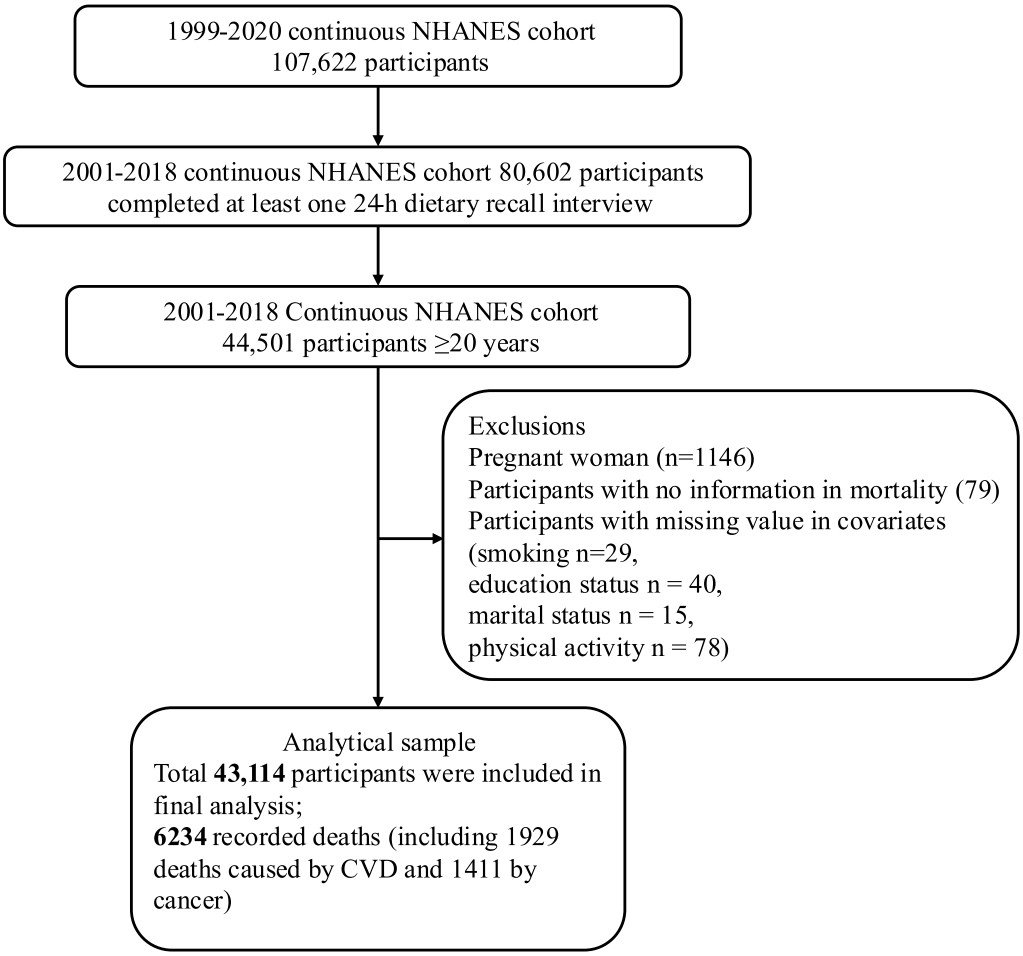

eFigure 1. Flowchart diagram of enrolled participants.
NHANES: National Health and Nutrition Examination Survey; CVD: cardiovascular disease

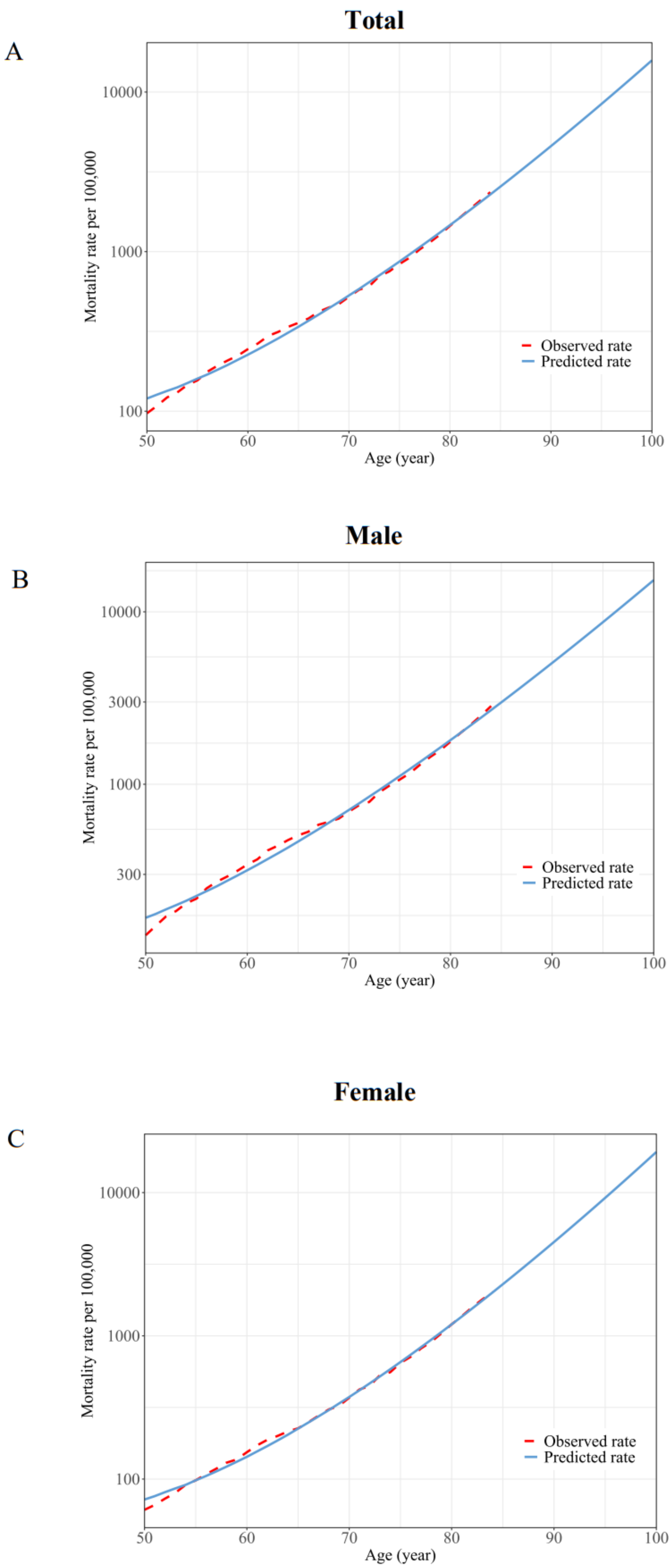

eFigure 2. Observed and predicted cardiovascular disease mortality rates of US population in 2019.
Predicted rates were assessed by Poisson model log-linear age + age^2.

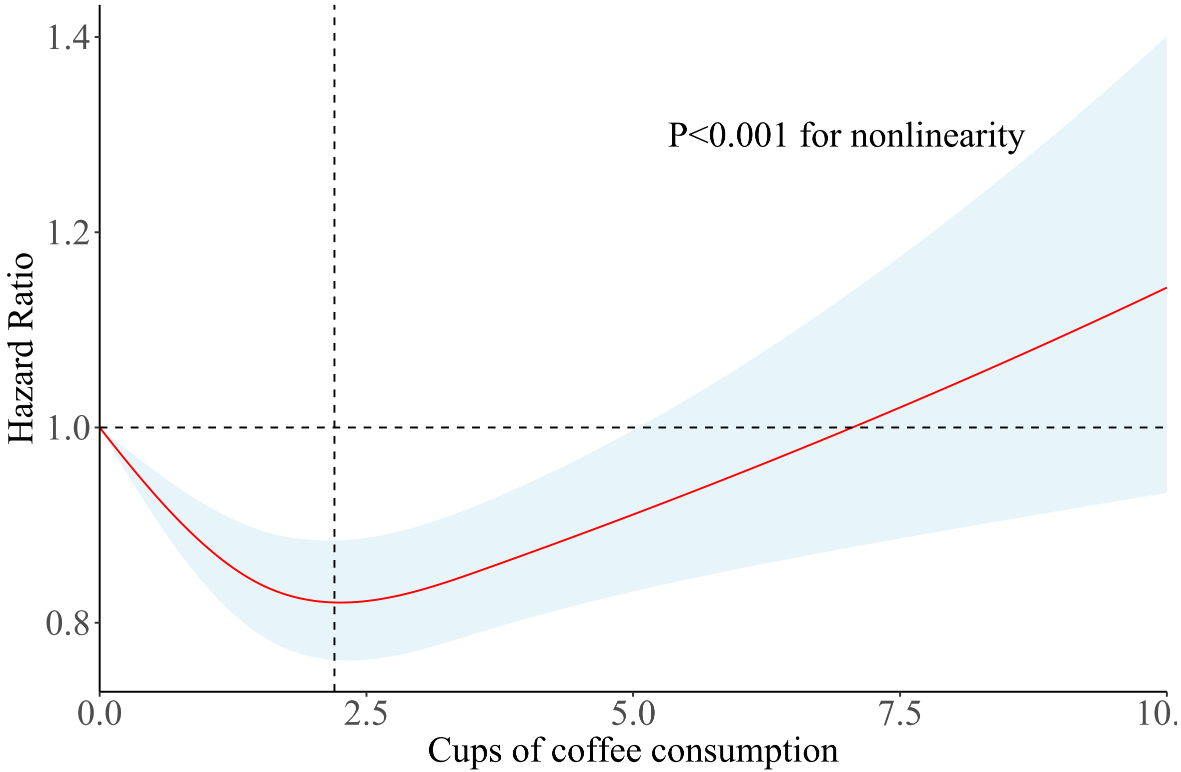

eFigure 3. Restricted cubic spline fitting for the association between tea consumption with all-cause mortality.
Red curve are multivariable adjusted hazard ratios, with light blue area showing 95% confidence intervals derived from restricted cubic spline regressions with three knots. Reference line for no association is indicated by the horizontal dash line at a hazard ratio of 1.0. Vertical dash line is located at 2.2 cups of coffee consumption, indicating the lowest hazard ratio (0.82).

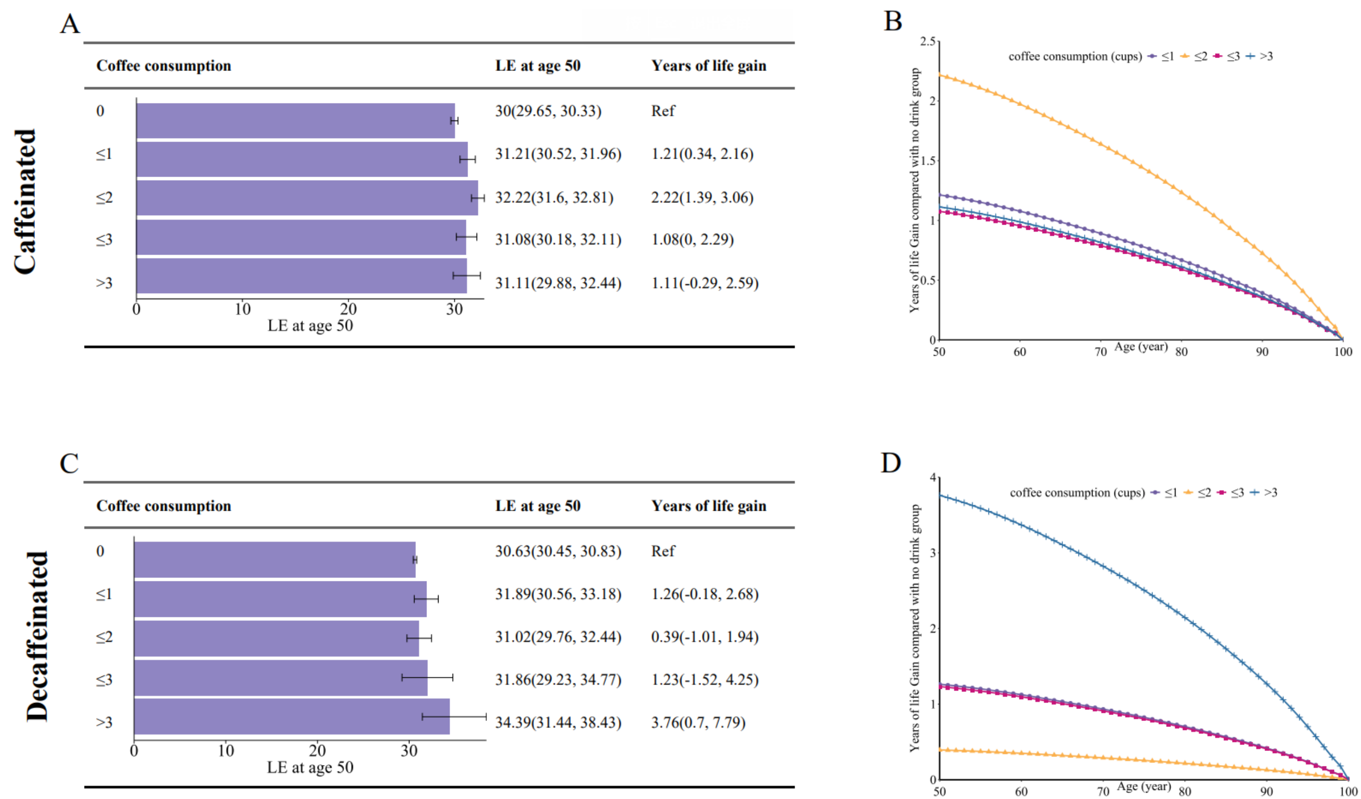

eFigure 4. Estimates of cumulative survival time from 50 years of age onward among participants with different levels of coffee consumption by caffeine status.

Abbreviations: LE, life expectancy; Ref, reference.

Life expectancy at age 50 according to coffee consumption levels in caffeinated coffee group (A), and decaffeinated or unknown coffee group (C). Life-years gain from other groups versus none consumption group from 50 to 100 years of age in caffeinated coffee group (B), and decaffeinated or unknown coffee group (D). The group of ≤2 represents 1 to ≤2 cups/day, and the group of ≤3 represents 2 to ≤3 cups/day.

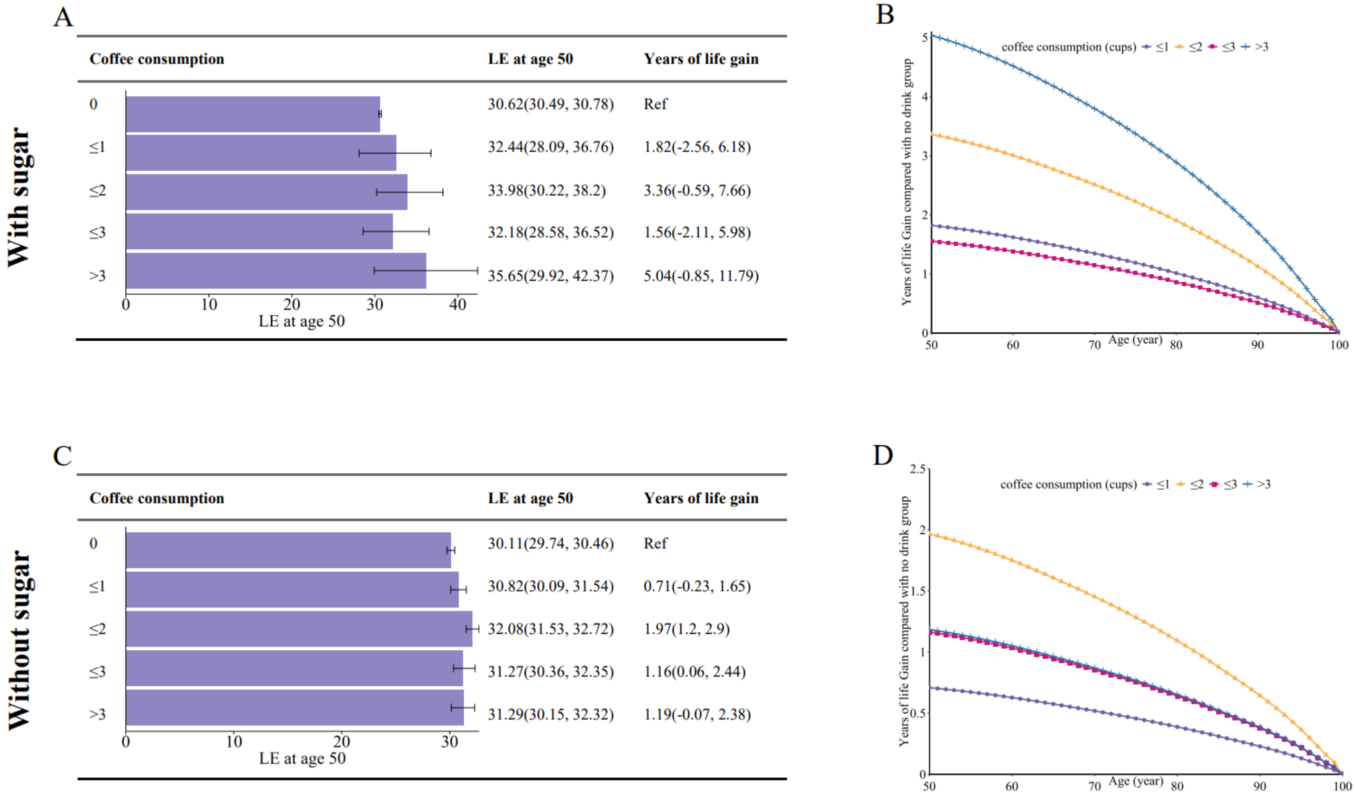

eFigure 5. Estimates of cumulative survival time from 50 years of age onward among participants with different levels of coffee consumption by sugar addition status.

Abbreviations: LE, life expectancy; Ref, reference.

Life expectancy at age 50 according to coffee consumption levels in with sugar group (A), and without sugar group (C). Life-years gain from other groups versus none consumption group from 50 to 100 years of age in with sugar group (B), and without sugar group (D). The group of ≤2 represents 1 to ≤2 cups/day, and the group of ≤3 represents 2 to ≤3 cups/day.

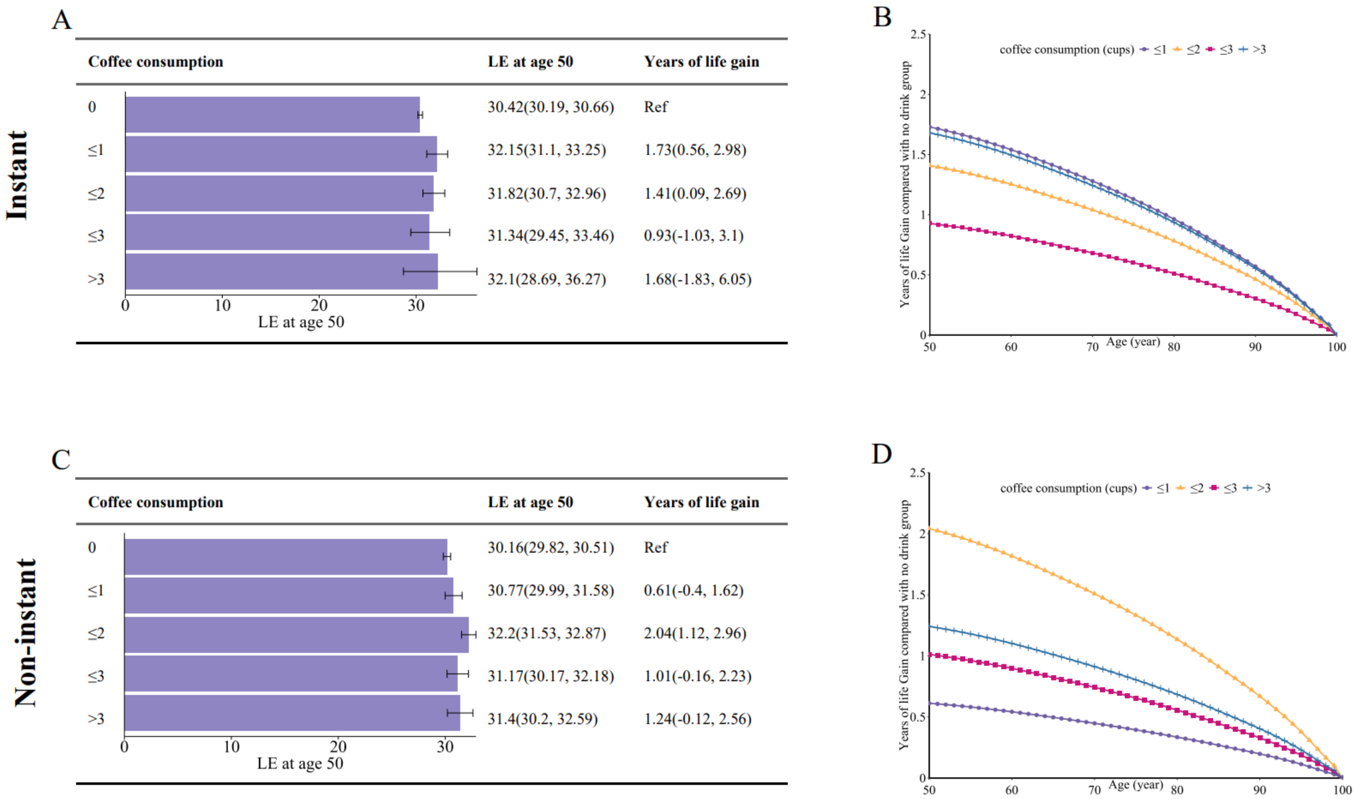

eFigure 6. Estimates of cumulative survival time from 50 years of age onward among participants with different levels of coffee consumption by preparation method.

Abbreviations: LE, life expectancy; Ref, reference.

Life expectancy at age 50 according to coffee consumption levels in instant coffee group (A), and non-instant coffee group (C). Life-years gain from other groups versus none consumption group from 50 to 100 years of age in instant coffee group (B), and non-instant coffee group (D). The group of ≤2 represents 1 to ≤2 cups/day, and the group of ≤3 represents 2 to ≤3 cups/day.
